# A meta-review of standard polysomnography parameters in Rett Syndrome

**DOI:** 10.3389/fneur.2022.963626

**Published:** 2022-09-20

**Authors:** Xin-Yan Zhang, Karen Spruyt

**Affiliations:** Université de Paris, INSERM NeuroDiderot, Paris, France

**Keywords:** Rett Syndrome, sleep, polysomnography, electroencephalogram (EEG), sleep disordered breathing (SDB)

## Abstract

Rett Syndrome (RTT, OMIM 312750), a unique rare neurodevelopmental disorder, mostly affects females and causes severe multi-disabilities including poor sleep. This meta-analysis systematically reviewed the polysomnographic (PSG) data of individuals with RTT on both sleep macrostructure and sleep respiratory indexes and compared them to literature normative values. Studies were collected from PubMed, Web of Science, PsycINFO, Ebsco, Scopus, and Cochrane Library till 26 April 2022. Across 13 included studies, the 134 selected RTT cases were mostly females being *MECP2* (*n* = 41) and *CDKL5* (*n* = 4) positive. They were further stratified by gene, age, and clinical features. Findings of comparison with literature normative values suggested shorter total sleep time (TST) and sleep onset latency (SOL), twice as long wake after sleep onset (WASO) with lower sleep efficiency (SEI) in RTT, as well as increased non-rapid eye movement stage 3 (stage N3) and decreased rapid eye movement sleep. Based on limited data per stratifications, we found in RTT cases <5 years old lower stage N3, and in RTT cases >5 years old less WASO and more WASO in the epileptic strata. However, meta-results generated from studies designed with comparison groups only showed lower stage N1 in RTT than in healthy comparison, together with similar SEI and stage N3 to primary snoring subjects. For sleep respiratory indexes, severe disordered sleep breathing was confirmed across roughly all RTT strata. We are the first study to meta-analyze PSG data of subjects with RTT, illustrating shorter TST and aberrant sleep staging in RTT that may vary with age or the presence of epilepsy. Severe nocturnal hypoxemia with apneic events was also demonstrated. More studies are needed to explore and elucidate the pathophysiological mechanisms of these sleep findings in the future.

**Systematic review registration:**

https://www.crd.york.ac.uk/prospero/display_record.php?RecordID=198099, identifier: CRD 42020198099.

## Introduction

Rett Syndrome (RTT, OMIM 312750) is a rare neurodevelopmental disorder with an approximate incidence of 1/10,000 in women (1). Classic RTT is characterized by stagnation and regression following a 6- to−18 month near-normal developmental period. Prominent features of RTT include loss of acquired hand skills and spoken language, the appearance of stereotypic movements, and gait or motor disabilities (2–4). Atypical RTT is symptomatically varied and named accordingly, such as preserved speech variant (PSV), early seizure onset variant (ESV), congenital variants (CV), and not otherwise specified atypical RTT (NOS-ARTT) (1). The causes of RTT have been strongly linked to the mutations in the gene encoding methyl-CpG-binding protein-2 (*MECP2*) on chromosome Xq28 (5). Other genetic candidates identified in RTT variants encode forkhead box protein G1 (*FOXG1*) on chromosome 14q13 (6) and cyclin-dependent kinase-like 5 (*CDKL5*) on chromosome Xq22.13 (7, 8). Hence, RTT is mainly found in girls. Over the years, diagnosis of RTT is primarily based on the presence or absence of criteria related to their cardinal clinical features (1, 2, 9, 10). Other signs also described in the RTT clinical profile include growth retardation, scoliosis, impaired sleep pattern, epilepsy, breathing disturbance, and autonomic abnormalities.

Previous studies (11–14) reported problematic sleeping in RTT such as frequent night waking [i.e., > 80% (15)] or night laughing [i.e., 77% (16)]. In fact, impaired sleep pattern was added to the supportive criteria since 2002 (9, 17, 18). In recent years, polysomnographic (PSG) studies in RTT have predominantly sought to record and describe sleep (19–22) from a clinical perspective. Regarding the sleep macrostructure, previous studies revealed prominently reduced rapid eye movement (REM) sleep and poor sleep efficiency in RTT (20, 23, 24). Although the breathing disturbances during wake state are well-recognized (25, 26), the findings during the sleep phase were inconsistent (19–21, 27), such that early studies reported normal sleep breathing pattern in RTT.

There is no meta review on PSG of RTT to date. We aimed to summarize the PSG parameters of sleep structure and sleep breathing events in RTT and to assess their differences when compared to normative values from a typically developing (TD) population reported in the literature. We explored additional analyses with respect to RTT features such as genes, age, and the presence of certain clinical features when reported to further adequately document the sleep characteristics in RTT.

## Methods

We followed the PRISMA 2009 reporting guideline (28) for this meta-review, which was registered in PROSPERO (CRD 42020198099). The quality of each study was scored via the Study Quality Assessment Tools of the National Institutes of Health (NIH) (29) by both authors. This tool applies to several study designs. We followed the same approach as published (30, 31). Study quality was regrouped on four domains: “study population, definition and selection,” “soundness of information,” “analysis, comparability, and outcomes,” and “interpretation and reporting,” and evaluated as poor, fair, and good. Disagreement in selection, extraction, and quality scoring was resolved by discussion.

### Search strategy and selection criteria

**Figure 1:**
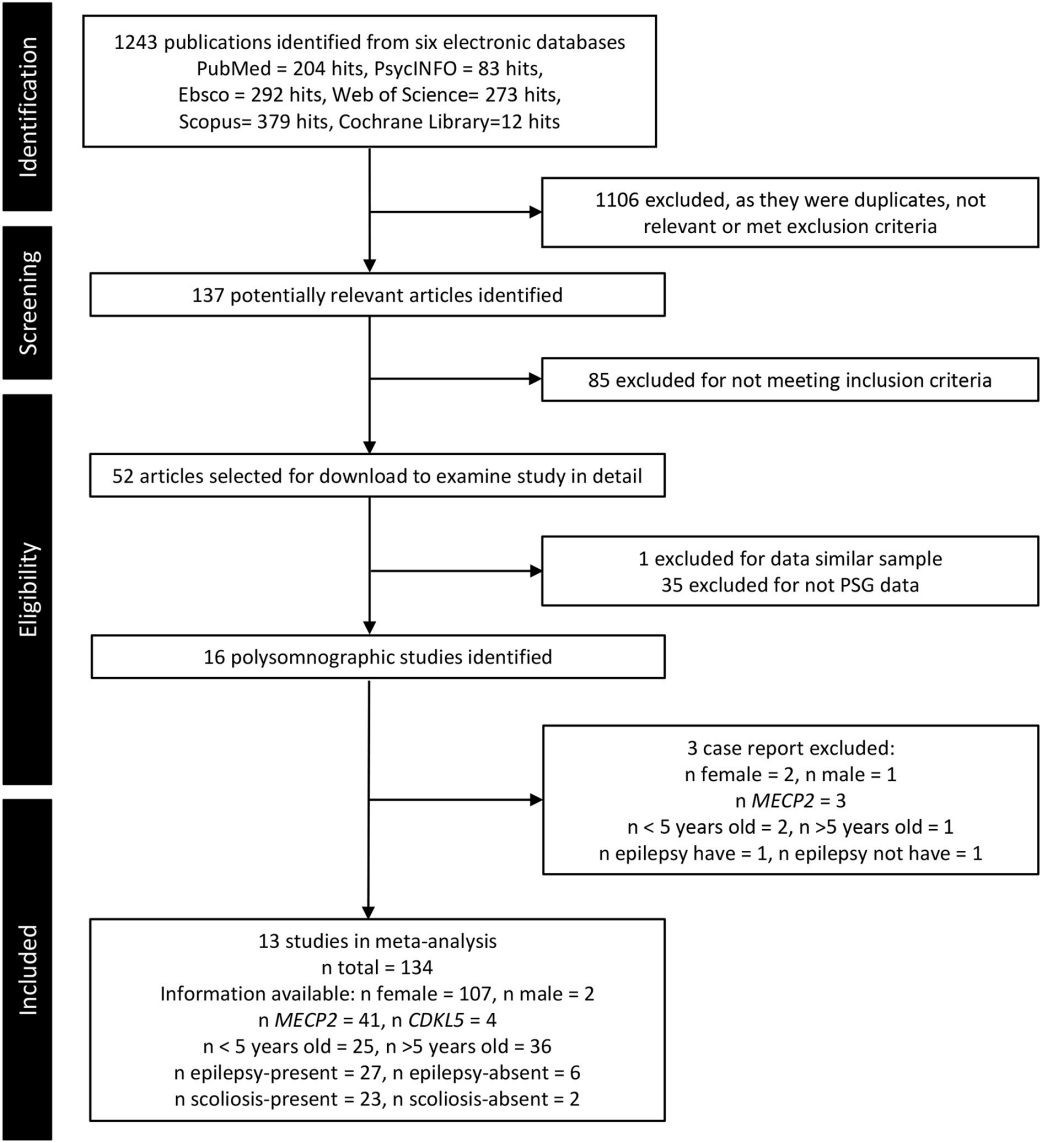
Flowchart of article selection up to date of 26 April 2022.

A systematic search was performed in PubMed, Web of Science, PsycINFO, Ebsco, Scopus, and Cochrane Library to 26 April 2022 (Figure 1) with the search terms: “Sleep AND Rett Syndrome” (see Supplementary material S1 for more details). Both authors screened and selected PSG (-related) studies on RTT individuals.

Studies were selected when fulfilling the following criteria: (1) original articles published in peer-reviewed journals; (2) RTT clinical or genetic diagnosis reported; (3) PSG data on sleep macrostructure and respiratory parameters (e.g., EEG spectrum analysis on sleep macrostructure was also included) printed numerically or graphically, which could be measured as numerical data. No time limitations or study design restrictions were applied. Studies would be excluded if on animal research (32–46) or if participants were RTT individuals with other central nervous system complications (e.g., neurofibroma) (47) or sleep intervention [e.g., PSG data from RTT cases after adeno(tonsil)ectomy surgery (A&T) history].

### Data collection and analysis

Demographic and methodologic information of studies including authors, sample characteristics, study design, PSG application, and conclusion were extracted.

Next, conventional PSG parameters were extracted and categorized into similar scoring methods (18). The definitions are listed in Table 1. For the studies that reported such information graphically, we measured and transferred data into numbers by a WebPlotDigitizer.

**Table 1:** PSG parameters and their definition in this study.

**Abbreviation**	**PSG parameter**	**Definition**
TST	Total sleep time	The time from sleep onset to the end of the final sleep epoch minus time awake
SOL	Sleep onset latency	Time from lights out to sleep onset
WASO	Wake after sleep onset	The time spent awake between sleep onset and end of sleep
SEI	Sleep efficiency	The ratio between total sleep time and time from lights out in the evening to lights on in the next morning expressed as a percentage
Stage N1	Non-rapid eye movement sleep stage 1	The amount of time in non-rapid eye movement sleep stage 1 per TST expressed as a percentage
Stage N2	Non-rapid eye movement sleep stage 2	The amount of time in non-rapid eye movement sleep stage 2 per TST expressed as a percentage
Stage N3	Non-rapid eye movement sleep stage 3	The amount of time in non-rapid eye movement sleep stage 3 per TST expressed as a percentage Stage N3 also includes stage N4 if reported separately, or defined as slow wave sleep (SWS)
REM	Rapid eye movement sleep	The amount of time in REM sleep per TST expressed as a percentage
AHI	Apnea/hypopnea index	The number of apnea and hypopnea events per hour of TST, normal value ≤ 1/h
OAHI	Obstructive apnea hypopnea index	The number of obstructive apneas and hypopneas per hour of TST
ODI	Oxygen desaturation index	The number of episodes of oxygen desaturation per hour of TST, with oxygen desaturation defined as a decrease in blood oxygen saturation (SpO_2_) to lower than 3% below baseline
SpO_2_% mean	Mean O_2_ saturation	Mean oxygen saturation
SpO_2_% nadir	Minimal O_2_ saturation	Minimal oxygen saturation

All available data were organized as the number of subjects (n), mean, and standard deviation (SD) for meta-analysis. In the publications reporting on cases series, sample mean and SD were calculated to represent the total group, or they were reported in subgroups when stratification was possible. Incompletely reported data and single case studies were excluded from the meta-analysis.

### Statistical analysis

Meta-analysis was performed in software Statistica TIBCO Software Inc. (48) version 13 and Meta-analysis with Comprehensive Meta-Analysis version 3.3.070 (Biostat, Englewood, NJ). Our approach consists of two parts.

In the first part, we summarized the extracted data to generate an average reference for RTT, i.e., the effect size (ES) is a pooled mean. These pooled means were subsequently compared to normative values of a TD population from the literature (49, 50) by a standardized mean difference test (SMD_*TD*_).

In the second part, in those studies that compared RTT samples to a comparison group, we performed SMD_*comparison*_ as ES on the summarized data.

For all the meta-analyses, random effects models were chosen, and the ES was illustrated by forest plots, with the size of the gray square showing the relative weight and the extent representing the 95% confidence intervals (95% CI). Particularly for the forest plots including the SMD_*TD*_, the range of normative values will be displayed as reference lines in red. Regarding the inconsistency across studies, Q-test and *I*^2^ (i.e., roughly 0 ≤ *I*^2^ ≤ 40%: might not be important, 40% < *I*^2^ ≤ 75%: may represent moderate heterogeneity, *I*^2^ > 75%: considerable heterogeneity) were applied to assess heterogeneity. The variance of the ES across the population of studies was reported by Tau^2^ (τ^2^). We performed a sensitivity analysis on RTT characteristics (e.g., gene, age, and the presence of certain clinical features). To test the robustness of our findings, Begg's correlation rank test was used for assessing the risk of publication bias. For all statistical analyses, *P* < 0.05 was set for statistical significance.

## Results

### Study selection and characteristics

Thirteen articles reported PSG data of subjects with RTT and fulfilled the criteria for our meta-review (Figure 1). Their summary of study design, participants, sleep assessment, NIH quality score, and the general conclusion is presented in Table 2. These 13 studies were published from 1985 to 2019 from six countries, with the majority being from the United States of America (i.e., six). Regarding the study design, seven case series and six observational studies with a cross-sectional design were included. Only seven studies reported specific diagnostic approaches, clinically and/or genetically. Regarding the sleep assessment, PSG (i.e., in 11 studies), video-polygraph (i.e., in one study), and EEG (i.e., in one study) were found, of which five followed the guidelines of American Academic Sleep Medicine (18) and two followed Rechtschaffen and Kales (58) scoring.

**Table 2:** Summary of included studies.

**Author**	**Country**	**Gender: *n***	**Age at PSG mean ±SD [range], y**	**Gene (n if not all)**	**Phenotype, Clinical stage (*n*)**	**Diagnostic methodology**	**Sleep assessment tool**	**Sleep scoring guideline**	**Sleep abnormalities**	**Type of Study**	**NIH quality assessment**	**Conclusion**
Sarber et al. (51)	United States of America	M: 2 F: 11	10.3 ± 4.9, [2.6–17.4]	*MECP2* (11)	Classic	C, G	PSG	AASM 2007-2017	SDB, sleep structure	Cross sectional retrospective data collection	Fair	Snoring and witnessed apneas were the most common complaints in RTT sleep
Amaddeo et al. (27)	France	F: 12^a^	9.3 ± 2.9 [6–16]	*MECP2* (11)		G	PSG	AASM 2007	SDB, sleep structure	Cross sectional prospective data collection	Poor	RTT have poor sleep quality with alterations in slow wave and REM
Bassett et al. (21)	United States of America	12^b^	7.8 ± 4.9, [1.9–17.6]				PSG		SDB	Case-series^g^	Fair	Respiratory abnormalities during sleep showed variability in RTT
Ammanuel et al. (52)	United States of America	RTT: F: 10 Control^c^: F: 15	RTT: 6.3 ± 2.1, [3.6–9.9] Control: 5.7 ± 1.8, [3–8]	*MECP2*			PSG	AASM 2007	EEG, sleep structure	Cross sectional retrospective data collection	Fair	SWS deficits such as fewer SWS cycles, heightened delta power in RTT
Carotenuto et al. (23)	Italy	RTT: 13 Control: 40	RTT: 8.1 ± 1.4 Control: 8.2 ± 1.0		Classic, III or IV		PSG	Miano S & American Thoracic Society 1996	SDB, sleep structure	Cross sectional prospective data collection	Fair	RTT group shows a great impairment in sleep macrostructure and sleep respiratory parameters
Hagebeuk et al. (22)	The Netherlands	F:10^d^	9.5 ± 8.8 [3–33]	*MECP2* (9)	III (9), IV (1)	C (9), G	PSG	AASM 2007	SDB	Case-series	Fair	Respiratory disturbances were present in all RTT cases
Hagebeuk et al. (53)	The Netherlands	F:4	6.5 ± 5.8 [2–15]	*CDKL5* (4)		G	PSG	AASM 2007	SDB, sleep structure	Case-series	Fair	Low REM, frequent arousals (not caused by apneas/seizures) and low SEI were present in *CDKL5* cases
Schluüter et al. (54)	Germany	F:2	13 ± 5.7 [9, 17]				PSG^f^	Schlüter 1993	SDB, EEG, sleep structure	Case-series	Fair	Sleep breathing disturbance was only seen in older RTT case
Marcus et al. (19)	United States of America	RTT: F: 30 Control^e^: F: 30	RTT: [1–17]; Control: [1–32]		II (1), III (24), IV (5)	C (10)	PSG	Rechtschaffen and Kales 1968	SDB, sleep structure	Cross sectional prospective data collection	Poor	RTT had similar sleep architecture and SEI from control group. Brainstem control of ventilation was normal in RTT
Segawa et al. (55)	Japan	F: 8^h^					PSG		EEG, sleep structure	Case-series	Fair	SWS(%) was within normal range in younger RTT group but decreased in older; REM was minimum increase with age
Aldrich et al. (56)	United States of America	F: 4	7.0 ± 3.0 [4–11]		III (4)	C (3)	PSG	Rechtschaffen and Kales 1968	EEG, sleep structure	Case-series	Fair	Spikes were most frequent during light NREM and all subjects had normal respiration during sleep in RTT
Glaze et al. (20)	United States of America	RTT: F: 11; Control: F&M 36	[2–15]				EEG		SDB, EEG, sleep structure	Cross sectional prospective data collection	Fair	Reduced REM, increased stage N2 and decreased sleep-latency in younger RTT group, reduced SEI in older. Only one case had obstructive apnea during REM sleep
Nomura et al. (57)	Japan	RTT: F: 5; Control: 1	RTT: 5.8 ± 4.4 [2–12] Control: 8				PSG	Segawa?	EEG, sleep structure	Case-series	Fair	Increasing in REM sleep as well as decreasing in SWS was along with age

a Five cases excluded for A&T history.

b Only extracted the first time PSG result in two cases, and two cases excluded for A&T history.

c Control group collected from age-matched girls clinically snoring but were otherwise healthy and have normal polysomnography studies.

d Only extracted the first time PSG result in one case, and two cases excluded for A&T history.

e Control group collected from age-matched female subjects with primary snoring (snoring without obstructive apnea or gas exchange abnormalities during sleep).

f Polygraphic technique included electroencephalogram, electro-oculogram, nasal airflow, thoracic and abdominal breathing movements, electrocardiogram, transcutaneous oxygen saturation.

g Correspondence letter.

h Eight RTT cases were recorded in 12 PSG recordings, of which six were done when the patients were under 5 years of age and the other six above the age of 5 years.

AASM, American Academy of Sleep Medicine; C, clinically; CDKL5, cyclin-dependent kinase-like 5; ECG, electrocardiography; EEG, electroencephalogram; F: female; M, male; G, genetically; MECP2, methyl-CpG-binding protein-2; NREM, non-rapid eye movement sleep; PSG, polysomnography; REM, rapid eye movement sleep; RTT, Rett Syndrome; stage N2, non-rapid eye movement sleep stage 2; SEI, sleep efficiency; SDB, sleep disordered breathing; SWS, slow wave sleep, and proportion of SWS pet total sleep time was represented as SWS (%).

We extracted in total 134 RTT cases aged from one to 33 years old and with sample sizes ranging from 2 to 30 subjects. Nine cases from three studies were excluded because their PSG was recorded after A&T surgery.

For the second part, five studies had a comparison group of a total of 122 subjects (note: one study with a single healthy child as a comparison group was not utilized for this meta calculation). Yet, two of those studies collected comparison subjects from a sample exhibiting primary snoring (i.e., *n* = 45).

### Study quality

**Figure 2:**
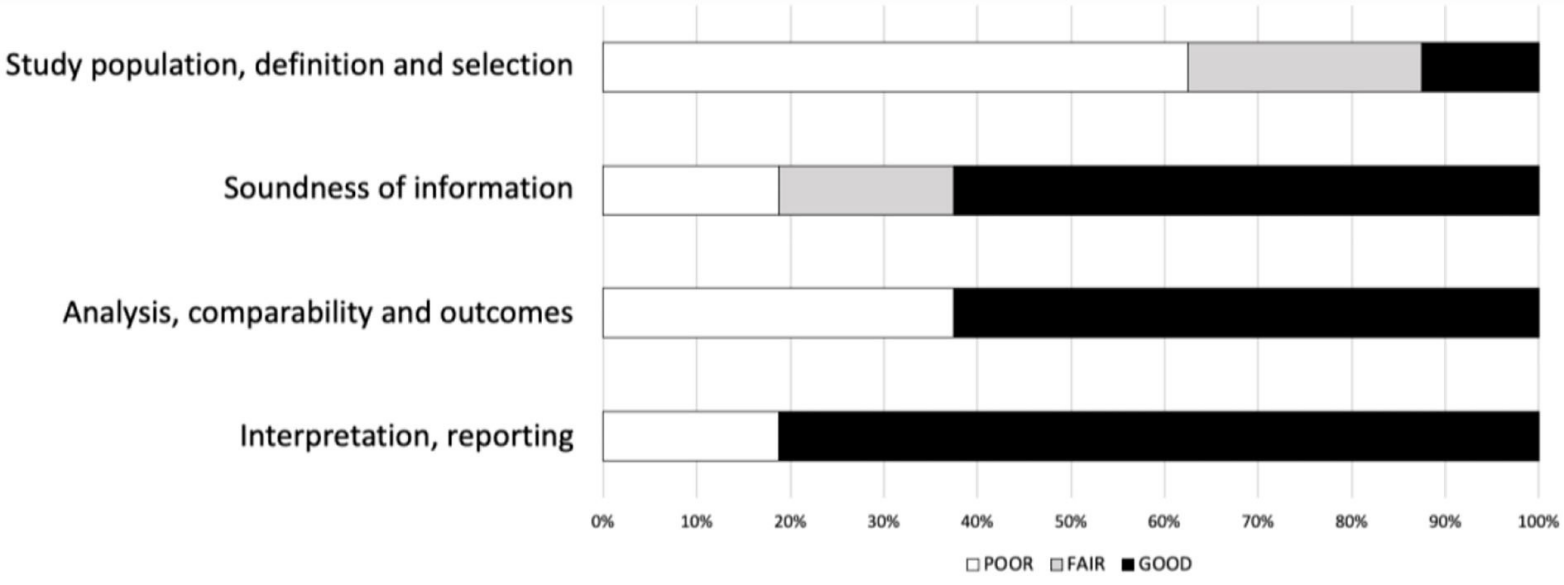
Risk of study quality bias (%). National Institutes of Health (NIH) quality assessment. The NIH quality assessment tool is applicable to different study designs and examines each study's internal validity by a set of items. For each study design, we subsequently regrouped items on four domains: “study population, definition, and selection,” “soundness of information,” “analysis, comparability, and outcomes,” and “interpretation and reporting.” A “poor” rating denotes that none of the items within the domain for the respective study design had an affirmative score; a “fair” rating is if less than half of the items were confirmed, and “good” is when more than half of the items were present. We report them as a proportion of the 13 studies examined to reflect the overall quality. Thus, for the 13 studies, a poor rating of more than 50% on the domain “study population, definition and selection” represents mediocre “reporting subject selection method, inclusion/exclusion criteria, sample characteristics etc”.

Most studies were rated as “fair” quality, with only 2/13 being of poor quality (Table 2). The cutback in quality was chiefly due to the inconsistent reporting of the diagnostic information (Figure 2).

### Meta-analysis part 1: Pooled mean of psg parameters in subjects with RTT and the comparison to literature normative values

Regarding the PSG data for subjects with RTT, sleep macrostructure parameters were collected from 11/13 studies and sleep-related breathing parameters from 9/13 studies. In addition to total group analysis, we were able to stratify the 134 RTT cases per gene (i.e., *MECP2* vs. *CDKL5*), age, and clinical features (i.e., presence or absence of epilepsy or scoliosis). Of note, the age cut-off being <5 years old vs. >5 years old was copied because several studies sub-grouped RTT cases at the age of 5 years.

#### Sleep macrostructure

Pooled means of sleep macrostructure parameters and results of SMD_*TD*_ are tabulated in Table 3. Forest plots are presented in Figures 1–8 in Supplementary material S2. RTT samples slept for about 7 h, resulting in a SEI of about 70%, whilst falling asleep took about 20 min, with wakefulness during the sleep period lasting to an hour or more. The distribution of sleep stages was as follows: stage N1 8%, stage N2 40%, stage N3 35%, and REM 15%.

**Table 3:** Pooled mean of sleep macrostructure in RTT subjects and the comparison with literature normative values for typically developing population (49) (Part 1).

**RTT stratification**		**Statistical analysis**		**TST**	**SOL**	**WASO**	**SEI**	**Stage N1**	**Stage N2**	**Stage N3**	**REM**
**TD population**	**Total group**	Normative values	Mean ± SD (n)	490.94 ± 26.56 (209)	25.81 ± 5.73 (209)	32.06 ± 14.27 (209)	89.53 ± 2.59 (209)	7.15 ± 0.52 (209)	39.69 ± 6.56 (209)	30.40 ± 4.84 (209)	21.32 ± 2.10 (209)
	**<** **5 years old**		Mean ± SD (n)	507.96 ± 30.81 (70)	29.97 ± 4.65 (70)	42.80 ± 22.13 (70)	87.50 ± 3.30 (70)	6.88 ± 0.47 (70)	32.54 ± 3.69 (70)	35.34 ± 2.81 (70)	23.74 ± 1.57 (70)
	**>** **5 years old**		Mean ± SD (n)	482.37 ± 22.08 (139)	23.71 ± 5.34 (139)	26.65 ± 4.50 (139)	90.56 ± 1.61 (139)	7.28 ± 0.54 (139)	43.30 ± 4.08 (139)	27.92 ± 3.49 (139)	20.11 ± 0.84 (139)
**RTT - Total group**		Pooled mean	n	76 (19, 20, 23, 27, 53, 54, 56)	75 (19, 20, 23, 51, 53, 56)	64 (19, 20, 23, 53, 54, 56)	99 (19, 20, 23, 27, 51–54, 56)	92 (19, 20, 23, 27, 51, 53, 56, 57)	92 (19, 20, 23, 27, 51, 52, 55–57)	114 (19, 20, 23, 27, 51–53, 55–57)	104 (19, 20, 23, 27, 51, 52, 55–57)
			ES ± SD (95% CI)	441.02 ±184.91 (399.45, 482.59)	19.34 ± 34.39 (11.56, 27.13)	66.06 ± 102.28 (41.00, 91.12)	74.22 ± 70.58 (60.31, 88.12)	8.11 ± 13.40 (5.37, 10.85)	40.16 ± 36.40 (32.72, 47.60)	35.47 ± 32.35 (29.53, 41.41)	16.38 ± 22.15 (12.12, 20.64)
			Heterogeneity	Q (6) = 42.13, *p* <0.01; 85.76%	Q (5) = 13.41, *p* = 0.02; 62.73%	Q (5) = 35.20, *p* <0.01; 85.79%	Q (8) = 782.91, *p* <0.01; 98.98%	Q (7) = 211.67, *p* <0.01; 96.69%	Q (7) = 115.84, *p* <0.01; 93.96%	Q (9) = 156.99, *p* <0.01; 94.27%	Q (8) = 769.71, *p* <0.01; 98.96%
			τ^2^	2097.67%	45.25%	604.40%	397.84%	11.84%	89.92%	71.00%	33.47%
			Test of overall effect	**20.79**, ***p*** **<** **0.01**	**4.87**, ***p*** **<** **0.01**	**5.17**, ***p*** **<** **0.01**	**10.46**, ***p*** **<** **0.01**	**5.81**, ***p*** **<** **0.01**	**10.58**, ***p*** **<** **0.01**	**11.71**, ***p*** **<** **0.01**	**7.54**, ***p*** **<** **0.01**
		Compared to TD	SMD*_*TD*_, p*	**0.51**, ***p*** **<** **0.01**	**0.35**, ***p*** **=** **0.01**	**−0.67**, ***p*** **<** **0.01**	**0.38**, ***p*** **<** **0.01**	−0.13, *p* = 0.30	−0.02, *p* = 0.86	**−0.26**, ***p*** **=** **0.03**	**0.38**, ***p*** **<** **0.01**
**RTT - Gene**	**MECP2**	Pooled mean	n	11 (27)	–	–	21 (27, 52)	11 (27)	11 (27)	21 (27, 52)	11 (27)
			ES ± SD (95% CI)	364.91 ± 105.17	–	–	54.08 ± 17.56 (46.56, 61.59)	1.64 ± 1.63	27.73 ± 15.82	53.06 ± 12.29 (47.80, 58.31)	12.64 ± 10.77
			Heterogeneity	–	–	–	Q (2) = 3.45, *p* = 0.18; 41.96%	–	–	Q (1) = 0.62, *p* = 0.43; 0%	-
			τ^2^	–	–	–	19.37%	–	–	0%	–
			Test of overall effect	–	–	–	**14.11**, ***p*** **<** **0.01**	–	–	**19.78**, ***p*** **<** **0.01**	–
		Compared to TD	SMD*_*TD*_, p*	–	–	–	**6.15**, ***p*** **<** **0.01**	–	–	**−3.85**, ***p*** **<** **0.01**	–
**RTT - Age**	**<** **5 years** **(2 – 5 years)**	Pooled mean	n	5 (53, 56)	5 (53, 56)	5 (53, 56)	9 (52, 53, 56)	8 (53, 56, 57)	8 (53, 56, 57)	14 (53, 55–57)	14 (53, 55–57)
			ES ± SD (95% CI)	524.13 ±214.61 (336.02, 712.24)	19.64 ± 31.79 (0, 47.50)	146.98 ± 262.07 (0, 376.70)	68.19 ± 35.52 (44.98, 91.40)	17.87 ± 15.95 (6.81, 28.92)	30.15 ± 7.52 (24.94, 35.37)	29.81 ± 21.14 (18.74, 40.88)	19.24 ±10.50 (13.74, 24.73)
			Heterogeneity	Q (1) = 10.71, *p* <0.01; 90.66%	Q (1) = 0.44, *p* = 0.51; 0%	Q (1) = 17.04, *p* <0.01; 94.13%	Q (2) = 34.60, *p* <0.01; 94.22%	Q (2) = 40.79, *p* <0.01; 95.10 %	Q (2) = 2.39, *p* = 0.30; 16.37%	Q (3) = 27.88, *p* <0.01; 89.24%	Q (3) = 14.69, *p* <0.01; 79.58%
			τ^2^	16785.24%	0%	25922.69%	393.72%	80.48%	4.77%	102.91%	23.31%
			Test of overall effect	**5.46**, ***p*** **<** **0.01**	1.38, *p* = 0.17	1.25, *p* = 0.21	**5.76**, ***p*** **<** **0.01**	**3.17**, ***p*** **<** **0.01**	**11.34**, ***p*** **<** **0.01**	**5.28**, ***p*** **<** **0.01**	**6.86**, ***p*** **<** **0.01**
		Compared to TD	SMD*_*TD*_, p*	−0.28, *p* = 0.55	**1.19**, ***p*** **=** **0.01**	**−1.60**, ***p*** **<** **0.01**	**1.63**, ***p*** **<** **0.01**	**−2.26**, ***p*** **<** **0.01**	0.57, *p* = 0.13	**0.63**, ***p*** **=** **0.03**	**1.02**, ***p*** **<** **0.01**
	**>** **5 years** **(6 – 17 years)**	Pooled mean	n	16 (27, 54, 56)	2 (56)	4 (54, 56)	22 (27, 52, 54, 56)	16 (27, 56, 57)	16 (27, 56, 57)	22 (27, 55–57)	22 (27, 55–57)
			ES ± SD (95% CI)	403.61 ± 220.46 (295.59, 511.64)	0.30 ± 0.28	18.51 ± 24.48 (0, 42.50)	70.17 ± 66.72 (42.29, 98.05)	11.52 ± 26.86 (0, 24.68)	35.43 ± 19.03 (26.10, 44.75)	36.04 ± 21.18 (27.19, 44.89)	18.52 ± 18.45 (10.81, 26.23)
			Heterogeneity	Q (2) = 9.35, *p* = 0.01; 78.62%	–	Q (1) = 1.00, *p* = 0.32; 0%	Q (3) = 57.70, *p* <0.01; 94.80%	Q (2) = 9.18, *p* = 0.01; 78.21%	Q (2) = 5.04, *p* = 0.08; 60.35%	Q (3) = 29.40, *p* <0.01; 89.80%	Q (3) = 19.17, *p* <0.01; 84.35%
			τ^2^	5714.03%	-	0%	641.02%	102.99%	41.32%	66.42%	51.50%
			Test of overall effect	**7.32**, ***p*** **<** **0.01**	-	1.51, *p* = 0.13	**4.93**, ***p*** **<** **0.01**	1.72, *p* = 0.09	**7.45**, ***p*** **<** **0.01**	**7.98**, ***p*** **<** **0.01**	**4.71**, ***p*** **<** **0.01**
		Compared to TD	SMD*_*TD*_, p*	**1.09**, ***p*** **<** **0.01**	-	**1.43**, ***p*** **=** **0.01**	**0.84**, ***p*** **<** **0.01**	−0.50, *p* = 0.06	**1.11**, ***p*** **<** **0.01**	**−0.97**, ***p*** **<** **0.01**	0.23, *p* = 0.31
**RTT- Clinical features**	**Epilepsy-present**	Pooled mean	n	19 (27, 53, 56)	8 (53, 56)	8 (53, 56)	19 (27, 53, 56)	19 (27, 53, 56)	19 (27, 53, 56)	19 (27, 53, 56)	19 (27, 53, 56)
			ES ± SD (95% CI)	477.98 ± 293.86 (345.85, 610.11)	10.04 ± 21.15 (0, 24.69)	127.24 ± 300.03 (0, 335.14)	75.16 ± 46.01 (54.48, 95.85)	15.43 ± 38.00 (0, 32.52)	36.48 ± 12.34 (30.93, 42.03)	27.33 ± 10.73 (22.50, 32.15)	11.86 ± 9.90 (7.41, 16.31)
			Heterogeneity	Q (2) = 25.37, *p* <0.01; 92.12%	Q (1) = 0.88, *p* = 0.35; 0%	Q (1) = 17.73, *p* <0.01; 94.36%	Q (2) = 25.47, *p* <0.01; 92.15%	Q (2) = 93.20, *p* <0.01; 97.85%	Q (2) = 1.28, *p* = 0.53; 0%	Q (2) = 0.95, *p* = 0.62; 0%	Q (2) = 2.79,			τ^2^	12233.32%	0%	21292.38%	305.86%	218.61%	0%	0%	4.41%
			Test of overall effect	**7.09, p** **<** **0.01**	1.34, *p* = 0.18	1.20, *p* = 0.23	**7.12**, ***p*** **<** **0.01**	1.77, *p* = 0.08	**12.89**, ***p*** **<** **0.01**	**11.10**, ***p*** **<** **0.01**	**5.22**, ***p*** **<** **0.01**
		Compared to TD	SMD*_*TD*_, p*	0.15, *p* = 0.53	**2.32**, ***p*** **<** **0.01**	**−1.70**, ***p*** **<** **0.01**	**1.09**, ***p*** **<** **0.01**	**−0.77**, ***p*** **<** **0.01**	0.45, *p* = 0.06	**0.55**, ***p*** **=** **0.02**	**2.75**, ***p*** **<** **0.01**

Meta-analysis is not applicable if there is only one study included for the parameter. Heterogeneity is presented as Q (df) = value, p-value; I^2^. Whereas τ^2^ stands for Between-study variance. The test of overall effect is printed as Z-value, p-value. The bold numbers are statistically significant. ES, effect size; MECP2, methyl-CpG-binding protein-2; REM, rapid eye movement sleep (%); RTT, Rett Syndrome; SD, standard deviation; SEI, sleep efficiency (%); SMD_*TD*_, standardized mean difference test with literature normative values from a TD sample, a negative SMD_*TD*_ indicates that the pooled mean in RTT > TD population, while a positive SMD_*TD*_ indicates that the pooled mean in RTT < TD population; SOL, sleep onset latency (min); stage N1, non-rapid eye movement sleep stage 1 (%); stage N2, non-rapid eye movement sleep stage 2 (%); stage N3, non-rapid eye movement sleep stage 3 (%); TD, typically developing; TST, total sleep time (min); WASO, wake after sleep onset (min); 95%CI, 95% confidence interval.

##### Total sample

In RTT total group SMD_*TD*_, we found significantly shorter TST, SOL, and particularly longer WASO. SEI was lower. Regarding the proportion of sleep stages, higher stage N3 and lower REM were seen in RTT.

##### Per gene strata

Yet based on fewer data, *MECP2* mutant cases showed a particularly lower SEI but also higher stage N3. In terms of other sleep macrostructure parameters in the *MECP2* stratum, the available data (k = 1, n = 11) was TST (364.91 ± 105.17 min.), stage N1 (1.64 ± 1.63 %), stage N2 (27.73 ± 15.82 %), and REM (12.64 ± 10.77 %). For the RTT cases in the *CDKL5* stratum (k = 1, n = 4) a TST (666.20 ± 110.40 min.), SOL (38.80 ± 63.20 min.), WASO (239.00 ± 99.00 min.), SEI (70.60 ± 11.70 %), stage N1 (25.00 ± 4.30 %), stage N2 (43.30 ± 14.90 %), stage N3 (22.80 ± 13.10 %), and REM (8.90 ± 6.60 %) was reported.

##### Per age strata

The SMD_*TD*_ results were discrepant between the two age strata except for SEI, i.e., in both age strata, SEI was lower. That is, in <5 years old stratum, longer WASO and shorter SOL were found, presenting further higher stage N1 but lower REM and stage N3, albeit the latter had a small SMD_*TD*_. Whilst in older ones, TST and particularly WASO were shorter but no data for SOL was available, and stage N1 and REM were not different from TD peers. However, a decreased stage N2 and an increased stage N3 were identified.

##### Per clinical features strata

We found the following only for the epilepsy-present stratum: the largest SMD_*TD*_ for REM but also lower SOL, more WASO and stage N1. Limited data was available to generate a pooled mean analysis for the following strata: i.e., epilepsy-absent RTT case (k = 1, *n* = 1) being TST (448.00 min), SEI (92.00 %), stage N1 (1.00 %), stage N2 (34.00 %), stage N3 (37.00 %), and REM (27.00 %); scoliosis-present RTT cases (k = 1, n = 12) with TST (349.75 ± 113.20 min), SEI (63.08 ± 22.19 %), stage N1 (2.50 ± 3.37 %), stage N2 (32.17 ± 21.54 %), stage N3 (52.50 ± 25.14 %), and REM (12.17 ± 10.40 %). No sleep macrostructure data was available for scoliosis-absent RTT cases.

#### Sleep respiratory

The pooled ES and the SMD_*TD*_ of the sleep respiratory parameters are all presented in Table 4 and Figures 9–13 in Supplementary material S2. AHI in RTT strata were all in the abnormal range.

**Table 4:** Pooled mean of sleep respiratory indexes in RTT subjects and the comparison with literature normative values for typically developing population (50) (Part 1).

**RTT stratification**		**Statistical analysis**		**AHI**	**OAHI**	**ODI**	**SpO_2_ mean**	**SpO_2_ nadir**
**TD population**	**Total group**	Normative values	Mean ± SD (n)	0.89 ± 0.84 (209)	0.00 ± 0.00 (209)	0.05 ± 0.05 (209)	97.00 ± 0.94 (209)	94.00 ± 1.05 (209)
	**<** **5 years old**		Mean ± SD (n)	1.77 ± 0.88 (70)	0.00 ± 0.00 (70)	0.10 ± 0.00 (70)	98.00 ± 0.00 (70)	92.71 ± 1.17 (70)
	**>** **5 years old**		Mean ± SD (n)	0.45 ± 0.32 (139)	0.00 ± 0.00 (139)	0.02 ± 0.05 (139)	97.65 ± 0.32 (139)	93.71 ± 0.24 (139)
**RTT - Total group**		Pooled mean	n	64 (21–23, 27, 51, 53)	35 (22, 27, 51)	35 (22, 23, 27)	42 (22, 23, 27, 53, 56)	53 (21, 22, 27, 51, 53, 56)
			ES ± SD (95% CI)	9.24 ± 29.60 (1.99, 16.48)	5.60 ± 11.83 (1.68, 9.52)	12.53 ± 11.42 (8.75, 16.32)	95.80 ± 2.83 (94.94, 96.65)	87.79 ± 13.72 (84.10, 91.49)
			Heterogeneity	Q (5) = 117.34, *p* <0.01; 95.74%	Q (2) = 5.24, *p* = 0.07; 61.80%	Q (2) = 2.50, *p* = 0.29; 19.90%	Q (4) = 21.50, *p* <0.01; 81.39%	Q (5) = 31.17, *p* <0.01; 83.96%
			τ^2^	66.36%	7.12%	3.22%	0.70%	15.68%
			Test of overall effect	**2.50**, ***p*** **=** **0.01**	**2.80**, ***p*** **=** **0.01**	**6.49**, ***p*** **<** **0.01**	**219.68**, ***p*** **<** **0.01**	**46.60**, ***p*** **<** **0.01**
		Compared to TD	SMD*_*TD*_, p*	**−0.58**, ***p*** **<** **0.01**	NA	**−2.92**, ***p*** **<** **0.01**	**0.84**, ***p*** **<** **0.01**	**1.00**, ***p*** **<** **0.01**
**RTT - Gene**	**MECP2**	Pooled mean	n	31 (22, 27, 51)	31 (22, 27, 51)	20 (22, 27)	20 (22, 27)	31 (22, 27, 51)
			ES ± SD (95% CI)	6.14 ± 12.36 (1.79, 10.49)	5.02 ± 9.76 (1.58, 8.45)	13.85 ± 32.60 (0, 28.15)	95.90 ± 2.84 (94.66, 97.15)	83.64 ± 13.01 (79.06, 88.22)
			Heterogeneity	Q (2) = 3.37, *p* = 0.19; 40.71%	Q (2) = 3.73, *p* = 0.15; 46.45%	Q (1) = 1.54 *p* = 0.21; 35.25%	Q (1) = 1.47, *p* = 0.23; 32.07%	Q (2) = 3.00, *p* = 0.22; 33.42%
			τ^2^	5.92%	4.22%	53.91%	0.26%	5.80%
			Test of overall effect	**2.76**, ***p*** **=** **0.01**	**2.86**, ***p*** **<** **0.01**	1.90, *p* = 0.06	**150.86**, ***p*** **<** **0.01**	**35.78**, ***p*** **<** **0.01**
		Compared to TD	SMD*_*TD*_, p*	**−1.18**, ***p*** **<** **0.01**	NA	**−1.79**, ***p*** **<** **0.01**	**0.90**, ***p*** **<** **0.01**	**2.19**, ***p*** **<** **0.01**
**RTT - Age**	**<** **5 years** **(1.9–5 years)**	Pooled mean	n	16 (21, 22, 51, 53)	6 (27, 51)	4 (22)	6 (22, 27)	16 (21, 22, 51, 53, 56)
			ES ± SD (95% CI)	1.89 ± 4.48 (0, 4.09)	0.81 ± 2.28 (0, 2.63)	6.65 ± 9.77	96.22 ± 2.84 (95.74, 96.70)	90.15 ± 6.68 (86.87, 93.42)
			Heterogeneity	Q (3) = 5.16, *p* = 0.16; 41.82%	Q (1) = 1.26, *p* = 0.26; 20.39%	–	Q (1) = 0.39, *p* = 0.53; 0%	Q (4) = 7.93, *p* = 0.09; 49.53%
			τ^2^	2.01%	0.79%	–	0%	6.30%
			Test of overall effect	1.68, *p* = 0.09	0.87, *p* = 0.39	–	**393.40**, ***p*** **<** **0.01**	**53.97**, ***p*** **<** **0.01**
		Compared to TD	SMD*_*TD*_, p*	−0.06, *p* = 0.83	NA	**−**	NA	**0.85**, ***p*** **<** **0.01**
	**>** **5 years** **(5 – 33 years)**	Pooled mean	n	34 (21, 22, 27, 51)	29 (22, 27, 51)	18 (22, 27)	20 (22, 27, 56)	34 (21, 22, 27, 51)
			ES ± SD (95% CI)	5.75 ± 15.39 (0.58, 10.92)	6.18 ± 12.60 (1.59, 10.76)	15.64 ± 31.33 (1.16, 30.11)	96.67 ± 1.02 (96.22, 97.11)	85.76 ± 21.12 (78.66, 92.86)
			Heterogeneity	Q (3) = 15.40, *p* <0.01; 80.52%	Q (2) = 5.54, *p* = 0.06; 63.87%	Q (1) = 1.56, *p* = 0.21; 35.70%	Q (2) = 1.53, *p* = 0.47; 0%	Q (3) = 25.89, *p* <0.01; 88.41%
			τ^2^	18.21%	10.19%	49.44%	0%	41.47%
			Test of overall effect	**2.18**, ***p*** **=** **0.03**	**2.64**, ***p*** **=** **0.01**	**2.12**, ***p*** **=** **0.03**	**425.78**, ***p*** **<** **0.01**	**23.67**, ***p*** **<** **0.01**
		Compared to TD	SMD*_*TD*_, p*	**−0.78**, ***p*** **<** **0.01**	NA	**−1.50**, ***p*** **<** **0.01**	**2.11**, ***p*** **<** **0.01**	**0.86**, ***p*** **<** **0.01**
**RTT – Clinical features**	**Epilepsy-present**	Pooled mean	n	22 (27, 51, 53)	19 (27, 51)	11 (27)	18 (27, 51, 56)	26 (27, 51, 53, 56)
			ES ± SD (95% CI)	6.29 ± 20.29 (0, 14.77)	7.53 ± 12.93 (1.72, 13.35)	30.14 ± 44.10	96.44 ± 0.70 (96.12, 96.76)	89.15 ± 10.55 (85.10, 93.21)
			Heterogeneity	Q (2) = 6.11, *p* = 0.05; 67.28%	Q (1) = 1.79, *p* = 0.18; 44.00%	–	Q (2) = 0.30, *p* = 0.86; 0%	Q (3) = 14.96, *p* <0.01; 79.95%
			τ^2^	33.50%	8.37%	–	0%	11.96%
			Test of overall effect	1.45, *p* = 0.15	**2.54**, ***p*** **=** **0.01**	–	**587.90**, ***p*** **<** **0.01**	**43.09**, ***p*** **<** **0.01**
		Compared to TD	SMD*_*TD*_, p*	**−0.87**, ***p*** **<** **0.01**	NA	–	**0.61**, ***p*** **=** **0.01**	**1.35**, ***p*** **<** **0.01**
	**Scoliosis-present**	Pooled mean	n	23 (27, 51)	23 (27, 51)	12 (27)	12 (27)	23 (27, 51)
			ES ± SD (95% CI)	12.36 ± 28.05 (0.90, 23.83)	7.96 ± 9.14 (4.23, 11.70)	27.79 ± 42.83	96.58 ± 2.15	85.27 ± 8.38 (81.84, 88.69)
			Heterogeneity	Q (1) = 1.42, *p* = 0.23; 29.59%	Q (1) = 0.62, *p* = 0.43; 0%	–	–	Q (1) = 0.12, *p* = 0.73; 0%
			τ^2^	33.96%	0%	–	–	0%
			Test of overall effect	**2.11**, ***p*** **=** **0.03**	**4.18**, ***p*** **<** **0.01**	–	–	**48.80**, ***p*** **<** **0.01**
		Compared to TD	SMD*_*TD*_, p*	**−1.32**, ***p*** **<** **0.01**	NA	–	–	**3.15**, ***p*** **<** **0.01**

Meta-analysis is not applicable if there is only one study included for the parameter, and SMD_*TD*_ test is not applicable if SD is 0 or the value not available. Heterogeneity is presented as Q (df) = value, p-value; I^2^. whereas τ^2^ stands for Between-study variance. The test of overall effect is printed as Z-value, p-value. Numbers in bold are statistically significant. AHI, apnea/hypopnea index per hour of TST (/h TST), normal value ≤ 1/h; ES, effect size; MECP2, methyl-CpG-binding protein-2; NA, not applicable; OAHI, obstructive apnea hypopnea index per hour of TST (/h TST); ODI, oxygen desaturation index per hour of TST (/h TST); RTT, Rett Syndrome; SD, standard deviation; SMD_*TD*_, standardized mean difference test with literature normative values from a TD sample, a negative SMD_*TD*_ indicates that the pooled mean in RTT > TD population, while a positive SMD_*TD*_ indicates that the pooled mean in RTT < TD population; SpO_2_ mean, Mean oxygen saturation (%); SpO_2_% nadir, Minimal oxygen saturation (%), normal range > 90%; TD, typically developing; TST, total sleep time; 95%CI, 95% confidence interval.

##### Total sample

In RTT total group, SMD_*TD*_ showed significantly higher AHI, ODI, and lower SpO_2_%.

##### Per gene, age, and clinical features strata

Comparable findings were seen in >5 years old, *MECP2*, epilepsy-present, and scoliosis-present strata. AHI and ODI in these strata were significantly higher than the TD population, whilst SpO2% mean and SpO_2_% nadir were lower. Particularly, the highest AHI was seen in the scoliosis-present stratum.

We should note the limited data for a pooled mean in epilepsy-present stratum for ODI (k = 1, *n* = 11, 30.13 ± 44.10 /h TST) and in scoliosis-present stratum for SpO_2_ mean (%) (k = 1, *n* = 12, 96.58 ± 2.15 %). Such limitation was more obvious in the other RTT strata, i.e., *CDKL5* mutant RTT cases (k = 1, *n* = 4) being AHI (1.48 ± 2.29 /h TST), SpO_2_% mean (96.30 ± 0.52 %), and SpO_2_% nadir (90.67 ± 3.21 %); epilepsy-absent RTT cases (k = 1, *n* = 5) being AHI (9.70 ± 7.61 /h TST), OAHI (8.46 ± 7.68 /h TST), and SpO_2_% nadir (87.20 ± 4.86 %) together with (k = 1, n = 1) ODI (2.00 /h TST) and SpO_2_% mean (98.00 %); and scoliosis-absent RTT cases (k = 1, n = 2) being AHI (4.20 ± 3.96 /h TST), OAHI (3.35 ± 3.46 /h TST), and SpO_2_% nadir (90.20 ± 1.27 %).

The difference test of OAHI and SpO2% mean in >5 years old stratum was not possible due to the SD of the literature normative value being “0,” but most RTT strata had OAHI >5/h TST.

### Meta-analysis part 2: Sleep macrostructure of subjects with RTT when compared to comparison groups as published in the reviewed papers

**Figure 3:**
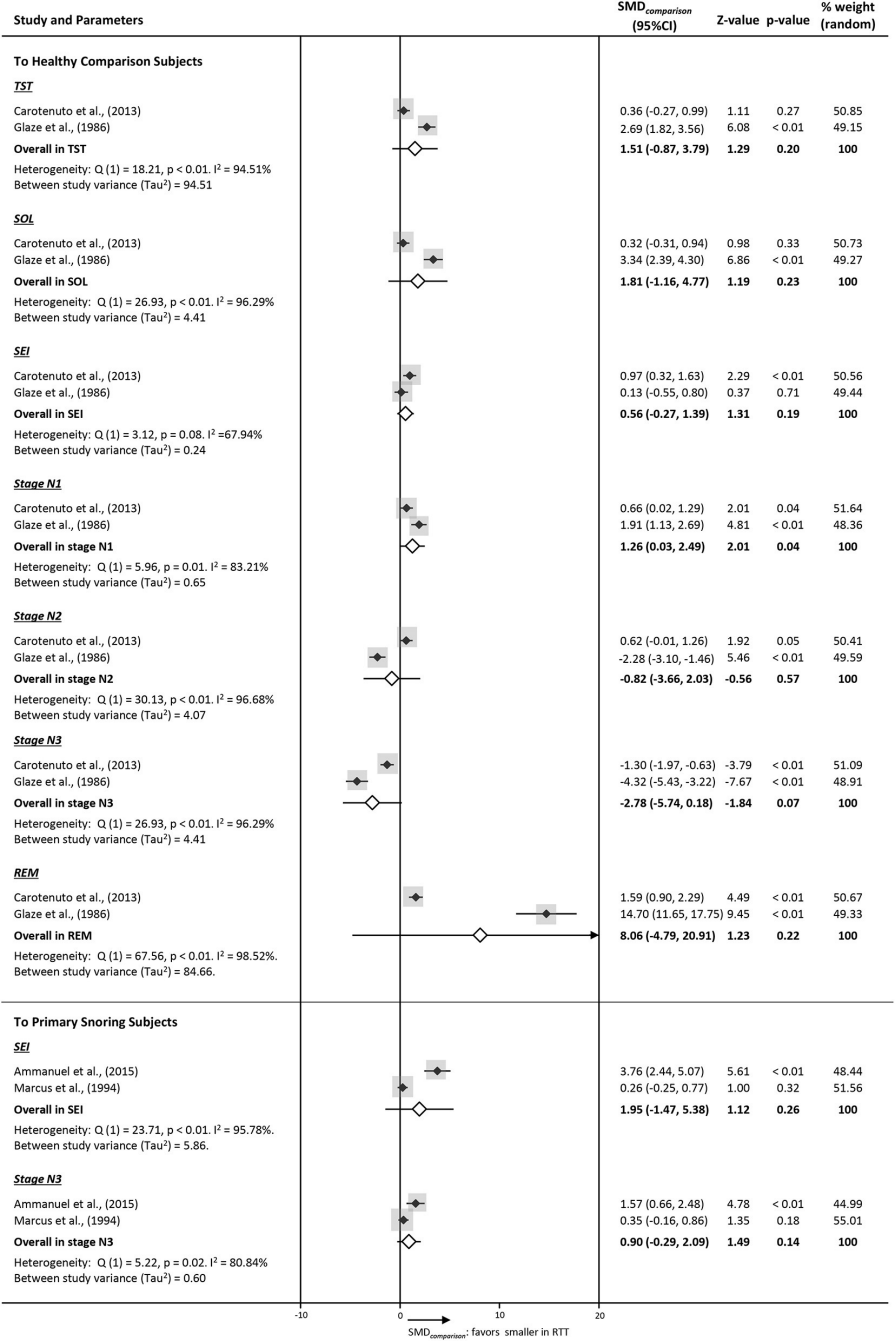
Forest plots of PSG parameters on sleep macrostructure of studies comparing RTT with a comparison group (SMD_*comparison*_). Diamonds indicate standard mean difference (SMD_*comparison*_) with a confidence interval of 95% (error bars 95% CI). The size of the gray square indicates the relative weight of the study on the combined ES.

We collected PSG data from four studies designed with comparison groups. Subjects with RTT recruited in these four studies were primarily classic phenotype under 15 years of age, and comparison groups were age-matched healthy (*n* = 76) and primary snoring subjects (*n* = 45) (e.g., k = 2 in each comparison) with limited stratification options. Forest plots are printed in Figure 3. Within the healthy comparison group, parameters of sleep macrostructure were extracted, and only stage N1 in RTT was found to be significantly lower. For the primary snoring group comparison, available data was limited to SEI and stage N3, both being non-significant.

### Publication bias

Publication bias was tested by Begg's correlation rank test and significance was only found for SpO_2_ nadir (%) (Supplementary material S3). After excluding this study, the findings were unaltered (*p* = 0.55).

## Discussion

We are one of the first systematic reviews providing average pooled sleep data of subjects with RTT, with further stratification per RTT-related genes, age, and the presence of certain clinical features. These data might be used as a reference regarding their sleep macrostructure and sleep respiratory events, with the note that eight studies included epilepsy-sensitive data. Compared to normative sleep study values, disrupted sleep in subjects with RTT can be chiefly characterized by increased WASO, prolonged stage N3 sleep, and attenuated REM sleep. Further, such SMD_*TD*_-based findings for RTT were somehow discrepant per stratifications of age and epilepsy. Principally for those younger than 5 years, more stage N1 and decreased stage N3, and for those older than 5 years shorter WASO, whereas for those with epilepsy especially reduced REM sleep was found. Contrariwise, yet given the limited number of studies included in the SMD_*comparison*_ analysis, only stage N1 was significantly lower than in healthy peers. Consequently, we may conclude poor sleep suggestive of variations according to certain RTT case features. Meta-findings further demonstrated severe nocturnal hypoxemia with apneic events.

### Sleep pattern and sleep stages

The sleep structure is deemed to be impaired in RTT. Firstly, we found consistently reduced SEI in RTT strata, which is further supported by shorter TST and longer WASO in some of our strata results. Such findings suggest poor sleep efficacy and continuity in RTT. Frequent (15, 59, 60) and longlasting (61) night wakenings have been repeatedly reported in previous RTT surveys. As known, when nocturnal sleep is disrupted, the sleep homeostatic system will promote sleep and compensate for the sleep loss (62), resulting in prolonged TST during the sleep “recovery” nights (63, 64). Regarding the TST in our SMD_*TD*_ results, we found that TST was contrariwise shortened in RTT total group and older stratum (i.e., 6–17 years), whilst not significantly different in the younger and epileptic RTT samples. For the TST reduction, integrating that previously we found that RTT cases with *CDKL5* mutation were sleeping significantly longer than those cases with *MECP2* mutation but with similar SEI and WASO (24), we may underline the genes-modulated pathological influence on their sleep regulation system. We believe that several other aspects should similarly be taken into consideration. Firstly, the sleep macrostructure is not identical across childhood. That is, the length of sleep time and its distribution within the 24-h period show an inverse relationship with age (65). A decreasing tendency in sleep duration in our reviewed sample, as shown in Table 3, might be assumed. Thus, our findings may suggest that the TST in RTT may decline more with age than TD. Secondly, sleep problems such as wakenings are common in young children (66) as well as in individuals with RTT (14), but in RTT cases they may persist. In fact, a modest peak of disorders of initiating and maintaining sleep in the 8–12 years old RTT age group was reported (14), potentially leading to shorter TST as found in our review. Thirdly, in terms of the presence of epilepsy, the drowsiness caused by epilepsy (67) or severe daytime somnolence due to antiepileptic drugs (AEDs) (68) may modify the sleep–wake cycle. We have to equally note here that the RTT samples in the younger and epilepsy-present strata were of a small sample size and from similar studies (53, 56). Lastly, the PSG recording time, and hence the potentially allowed time in bed and sleep duration, by convention, is being determined by a convenient wake-up timing on the next morning for sleep staff, which is acknowledged and likely discrepant from the usual sleep schedules of subjects with RTT. Alternatively, the “first night effect,” characterized as decreased TST, lower SEI, reduced REM, and longer REM latencies on the first PSG testing night (69, 70) might be considered as well to describe their poor sleep, and only one reviewed study (23) discussed this. Thus, a single night PSG recording during the nocturnal phase might be limitedly representative or may further aggrevate the alterations of sleep in RTT.

Although limited data on SOL was extracted, yet being another aspect of homeostatic recovery regulation, we found a consistent shortened SOL, which may reflect the increased sleep pressure, as reported before (24). Yet, it contradicts with our findings from sleep problem surveys where “difficulty falling asleep” was found in 60.3% of a *MECP2* RTT group (14).

Regarding the sleep stage distribution, results for stage N1 and stage N3 sleep were peculiar. For stage N1, the SMD_*TD*_ showed in the younger and epileptic strata a higher stage N1, but the SMD_*comparison*_ showed a decreased proportion (k = 2). Per the forest plot depicting stage N1, in three studies (53, 56, 57) stage N1 was relatively higher (see Figure 5 in Supplementary material S2), yet these studies did not report comparisons to a group and therefore were not included in the part 2 analysis. Further, when we screened the sample characteristics of these three studies, they chiefly represent samples within a 10-year age-range and with severe epilepsy or being treated with AEDs (53, 56) (i.e.: 5/8 of these epileptic RTT cases were samples for <5 years old stratum). Individuals with epilepsy usually have longer stage N1 sleep (71). Although epilepsy is prevalent in RTT, the incidence of epilepsy and the severity of seizure is thought to be milder in classic RTT phenotype than in certain atypical variants (72). In fact, only classic RTT cases were used in the comparison group studies, which could explain our discrepant stage N1 results.

Stage N2 showed a reduction in the older RTT stratum only. In healthy individuals, stage N2 has been reported to be significantly increased with age (65). We could not confirm this typical aging effect of stage N2 in RTT, neither here nor in our previous review (24) on literature case series data.

Though previous studies (23, 27, 51) and our SMD_*TD*_ illustrated a relative increased stage N3, the stage N3 by SMD_*comparison*_ to healthy subjects was not significant. Yet after stratification, we confirmed higher stage N3 in the *MECP2* RTT cases (i.e., excluding the *CDKL5* from the total sample), as previously illustrated (24). The *CDKL5* sample collected in this study was too small for meta-analysis, but we previously reported that they have less stage N3 sleep than *MECP2* mutant RTT cases (24). Another confounder in stage N3 sleep findings besides genes might be age-related alterations. That is, here we found that stage N3 was significantly lower in younger RTT cases but higher in the older ones. Such stage N3 alteration is opposite to the age-related proportional decline of the N3 stage in the general population as reported by Ohayon et al. (65). This finding may demonstrate the perturbed slow oscillations occurring in RTT further challenging their sleep stage transition from deeper sleep stages (73, 74).

In terms of REM sleep, reduced REM was reported as a characteristic in several sleep studies of RTT (20, 23, 51) and our case series review (24). We also found decreased REM in subjects with RTT, but could not confirm this in the >5 year-old stratum. Such a dissimilarity, particularly, in earlier studies as increased (75) or decreased (20) REM in older RTT cases have been reported, could be linked to the brainstem dysfunctioning toward sleep cycle generation given a dispersed age at onset. However, based on our previous case series data, REM does not change with chronological age (24), and intergrating with the decline of REM sleep proportion in a healthy population (65), we may assume that the REM in RTT may stagnate already early in the life.

### Sleep breathing

Although the breathing pattern in RTT seems more regular during nighttime than daytime, our study provided evidences supporting severe sleep-disordered breathing (SDB). During sleep, significantly more desaturations occur with steady hypoxemia. In fact, a wide spectrum of breathing irregularities during the wake-phase has been vividly described and discussed in RTT (76–78). Some studies (21, 53) likewise illustrated irregular sleep breathing patterns by central apnea, which may be due to immaturity of the respiratory control system (79). Based on plethysmography, dysregulation in the autonomic nervous system especially for younger RTT cases (80) was shown. Meanwhile, such sleep breathing irregularities may cause chronic hypoxemia during sleep as in our results, leading to reduced hypoxic sensitivity for chemoreceptor responding pathways in the brainstem.

In terms of the mechanism, both *Mecp2* and *Cdkl5* genetic mutations were proven to cause breathing abnormalities in RTT animal models, being more frequent during non-rapid eye movement sleep (NREM) in *Cdkl5* mutant mice, which worsens with age in *Mecp2* ones (81). The AHI is highly severe in those >5 years old, and with *MECP2*, epilepsy, and scoliosis in our meta-review. Regarding potentially involved molecules, such as GABA, brain-derived neurotrophic factor (BDNF) and monoaminergic modulators (76) have been suspected; however, the findings remain to be further clarified in human literature.

In view of the possible influence of SDB on the sleep macrostructre, our meta-analysis confirmed that RTT cases had similar SEI and stage N3 to primary snoring subjects (i.e., part 2 meta-analysis). But previous findings on sleep macrostructure differences between subjects with RTT and primary snoring subjects have been scant and inconsistent (19, 52) and consolidated in our meta-review. Thus, the impact of SDB on their sleep macrostructure is still a burning question.

Several limitations of our meta-review should be noted. Variable definitions of PSG parameters and the complexity of symptomatology and pathogenicity in RTT may have largely contributed to the heterogeneity among published studies. Although we did stratification per RTT-related genes, age, and the presence of certain clinical features, PSG data is indeed scant with regard to specific parameters (e.g., SOL), several strata (e.g., *CDKL5*, absence of epilepsy, and scoliosis), and more powerful study designs (e.g., with the control group). The age cut-off, although to a certain extent arbitrary, was copied from previous studies, allowing the analysis of a maximum amount of data per such stratum. Furthermore, we could not meta-analyze the data per cardinal RTT features due to the fact that studies did not report RTT samples following the guideline designed for cases with *MECP2* mutations (4). Cases herein may however be more severe (e.g., breathing, epilepsy) as being referred for a sleep study, and therefore a selection bias may exist. While the TD comparsion sample was all under 18 years of age, only one RTT case in our sample was 33 years old. Although different sleep scoring methods and reporting formats may lead to biases, each sleep stage scoring methodology is standardized (18, 58, 82, 83), and therefore should not lead to too discordant findings. Also, PSG studies of RTT cases with *FOXG1* mutation were not found. Thus, our review also underlined the scarcity of PSG investigation in RTT.

## Conclusion

The findings are based on a limited number of PSG studies, but in general increased WASO with more stage N3 sleep and less REM sleep was found. Yet, age and epilepsy might be potential moderators of NREM sleep proportional distributions, whereas they might be a mediator for REM sleep generation. During the nighttime, a hypoxic state with apneic events was demonstrated. Our findings may help to elucidate multifactorial pathomechanisms in this complex disease and may stimulate basic research on mechanistic pathways in existing RTT animal models.

## Data availability statement

The original contributions presented in the study are included in the article/Supplementary material, further inquiries can be directed to the corresponding author.

## Author contributions

KS conceived and planned the presented review. X-YZ executed the review and wrote the first draft. Both authors verified and discussed the results and contributed to the final manuscript.

## Conflict of interest

The authors declare that the research was conducted in the absence of any commercial or financial relationships that could be construed as a potential conflict of interest.

## Publisher's note

All claims expressed in this article are solely those of the authors and do not necessarily represent those of their affiliated organizations, or those of the publisher, the editors and the reviewers. Any product that may be evaluated in this article, or claim that may be made by its manufacturer, is not guaranteed or endorsed by the publisher.

## Supplementary material

Supplementary material S1 Search items applied up to date 26 April 2022.

Supplementary material S2 Figures 1–13: Forest plots of sleep macrostructure and sleep respiratory parameters in RTT (Part 1).

Supplementary material S3 Begg's correlation rank test for publication bias.
