# Supplementary material for: A meta-review of standard polysomnography parameters in Rett Syndrome
Source: Front Neurol. 2022 Sep 20;13:963626. doi: 10.3389/fneur.2022.963626 (PMC9530595; doi:10.3389/fneur.2022.963626)
Supplement: Supplementary material S1 — Search items applied up to date 26 April 2022. [file Data_Sheet_1.docx]

Supplementary Material

**Supplement 1: Search items of cut-off date 26 April 2022**

| PubMed (total of 204 original citation) | |
| --- | --- |
| Search #1 | (((((((((((((rett syndrome) OR Syndrome, Rett) OR Autism-Dementia-Ataxia-Loss of Purposeful Hand Use Syndrome) OR Autism-Dementia-Ataxia-Loss of Purposeful Hand Use Syndrome) OR (Autism, Dementia, Ataxia, and Loss of Purposeful Hand Use)) OR Rett Disorder) OR Rett's Disorder) OR Rett's Syndrome) OR Retts Syndrome) OR Syndrome, Rett's) OR Cerebroatrophic Hyperammonemia) OR Cerebroatrophic Hyperammonemias) OR Hyperammonemia, Cerebroatrophic) OR Hyperammonemias, Cerebroatrophic (identified 4,245 document citations) |
| Search #2 | Sleep (identified 260,261 document citations) |
| Search #3 | Search #1 AND # 2 (((((((((((((((rett syndrome) OR Syndrome, Rett) OR Autism-Dementia-Ataxia-Loss of Purposeful Hand Use Syndrome) OR Autism-Dementia-Ataxia-Loss of Purposeful Hand Use Syndrome) OR (Autism, Dementia, Ataxia, and Loss of Purposeful Hand Use)) OR Rett Disorder) OR Rett's Disorder) OR Rett's Syndrome) OR Retts Syndrome) OR Syndrome, Rett's) OR Cerebroatrophic Hyperammonemia) OR Cerebroatrophic Hyperammonemias) OR Hyperammonemia, Cerebroatrophic) OR Hyperammonemias, Cerebroatrophic)) AND sleep (identified 204 document citations) |
| Web of Science (total of 273 original citation) | |
| Set #1 | TOPIC: (rett syndrome) OR TOPIC: (Syndrome, Rett) OR TOPIC: (Autism-Dementia-Ataxia-Loss of Purposeful Hand Use Syndrome) OR TOPIC: (Autism Dementia Ataxia Loss of Purposeful Hand Use Syndrome) OR TOPIC: (Autism, Dementia, Ataxia, and Loss of Purposeful Hand Use) OR TOPIC: (Rett Disorder) OR TOPIC: (Rett's Disorder) OR TOPIC: (Rett's Syndrome) OR TOPIC: (Retts Syndrome) OR TOPIC: (Syndrome, Rett's) OR TOPIC: (Cerebroatrophic Hyperammonemia) OR TOPIC: (Cerebroatrophic Hyperammonemias) OR TOPIC: (Hyperammonemia, Cerebroatrophic) OR TOPIC: (Hyperammonemias, Cerebroatrophic) Indexes=SCI-EXPANDED, SSCI, A&HCI, CPCI-S, CPCI-SSH, BKCI-S, BKCI-SSH, ESCI, CCR-EXPANDED, IC Timespan=All years (identified 6,280 document citations) |
| Set #2 | TOPIC: (sleep) Indexes=SCI-EXPANDED, SSCI, A&HCI, CPCI-S, CPCI-SSH, BKCI-S, BKCI-SSH, ESCI, CCR-EXPANDED, IC Timespan=All years (identified 333,121 document citations) |
| Set #3 | #2 AND #1 Indexes=SCI-EXPANDED, SSCI, A&HCI, CPCI-S, CPCI-SSH, BKCI-S, BKCI-SSH, ESCI, CCR-EXPANDED, IC Timespan=All years (identified 273 document citations) |
| Scopus (total of 379 original citations) | |
| Search #1 | ( TITLE-ABS-KEY ( rett  AND syndrome )  OR  TITLE-ABS-KEY ( syndrome,  AND rett )  OR  TITLE-ABS-KEY ( autism-dementia-ataxia-loss  AND of  AND purposeful  AND hand  AND use  AND syndrome )  OR  TITLE-ABS-KEY ( autism  AND dementia  AND ataxia  AND loss  AND of  AND purposeful  AND hand  AND use  AND syndrome )  OR  TITLE-ABS-KEY ( autism,  AND dementia,  AND ataxia,  AND  loss  AND of  AND purposeful  AND hand  AND use )  OR  TITLE-ABS-KEY ( rett  AND disorder )  OR  TITLE-ABS-KEY ( rett's  AND disorder )  OR  TITLE-ABS-KEY ( rett's  AND syndrome )  OR  TITLE-ABS-KEY ( retts  AND syndrome )  OR  TITLE-ABS-KEY ( syndrome,  AND rett's )  OR  TITLE-ABS-KEY ( cerebroatrophic  AND hyperammonemia )  OR  TITLE-ABS-KEY ( cerebroatrophic  AND hyperammonemias )  OR  TITLE-ABS-KEY ( hyperammonemia,  AND cerebroatrophic )  OR  TITLE-ABS-KEY ( hyperammonemias,  AND cerebroatrophic ) )  (identified 5,901 document citations) |
| Search #2 | TITLE-ABS-KEY ( sleep )  (identified 313,174 document citations) |
| Search #3 | ( ( TITLE-ABS-KEY ( rett  AND syndrome )  OR  TITLE-ABS-KEY ( syndrome,  AND rett )  OR  TITLE-ABS-KEY ( autism-dementia-ataxia-loss  AND of  AND purposeful  AND hand  AND use  AND syndrome )  OR  TITLE-ABS-KEY ( autism  AND dementia  AND ataxia  AND loss  AND of  AND purposeful  AND hand  AND use  AND syndrome )  OR  TITLE-ABS-KEY ( autism,  AND dementia,  AND ataxia,  AND  loss  AND of  AND purposeful  AND hand  AND use )  OR  TITLE-ABS-KEY ( rett  AND disorder )  OR  TITLE-ABS-KEY ( rett's  AND disorder )  OR  TITLE-ABS-KEY ( rett's  AND syndrome )  OR  TITLE-ABS-KEY ( retts  AND syndrome )  OR  TITLE-ABS-KEY ( syndrome,  AND rett's )  OR  TITLE-ABS-KEY ( cerebroatrophic  AND hyperammonemia )  OR  TITLE-ABS-KEY ( cerebroatrophic  AND hyperammonemias )  OR  TITLE-ABS-KEY ( hyperammonemia,  AND cerebroatrophic )  OR  TITLE-ABS-KEY ( hyperammonemias,  AND cerebroatrophic ) ) )  AND  ( TITLE-ABS-KEY ( sleep ) )  (identified 379 document citations) |
| PsycINFO (total of 83 original citations) | |
| Search | Any Field: rett syndrome  AND  Any Field: sleep  Search Databases: APA PsycInfo, APA PsycArticles, APA PsycBooks, APA PsycExtra (identified 83 document citations) |
| EBSCO (MEDLINE) (total of 292 original citations) | |
| Search | Rett syndrome AND sleep  Interface - EBSCOhost Research Databases  Search Screen - Advanced Search  Database - MEDLINE;Academic Search Premier;Business Source Premier;ERIC;GreenFILE;Library, Information Science & Technology Abstracts;Newspaper Source;Regional Business News;Teacher Reference Center;European Views of the Americas: 1493 to 1750;eBook Collection (EBSCOhost);EBSCO eClassics Collection (EBSCOhost);OpenDissertations;The Belt and Road Initiative Reference Source;CINAHL Complete 292 (total of 292 original citations) |
| Cochrane Library (total of 12 original citations) | |
| Search | ("Rett syndrome"):ti,ab,kw AND (sleep):ti,ab,kw (total of 12 Trials matching) |
