# Supplementary material for: A meta-review of standard polysomnography parameters in Rett Syndrome
Source: Front Neurol. 2022 Sep 20;13:963626. doi: 10.3389/fneur.2022.963626 (PMC9530595; doi:10.3389/fneur.2022.963626)
Supplement: Supplementary material S2 — Figures 1–13: Forest plots of sleep macrostructure and sleep respiratory parameters in RTT (Part 1). [file Data_Sheet_2.pdf]

Supplementary 2: Figure 1. Forest plots of sleep macrostructure in RTT — Total sleep time (TST) (Part 1)

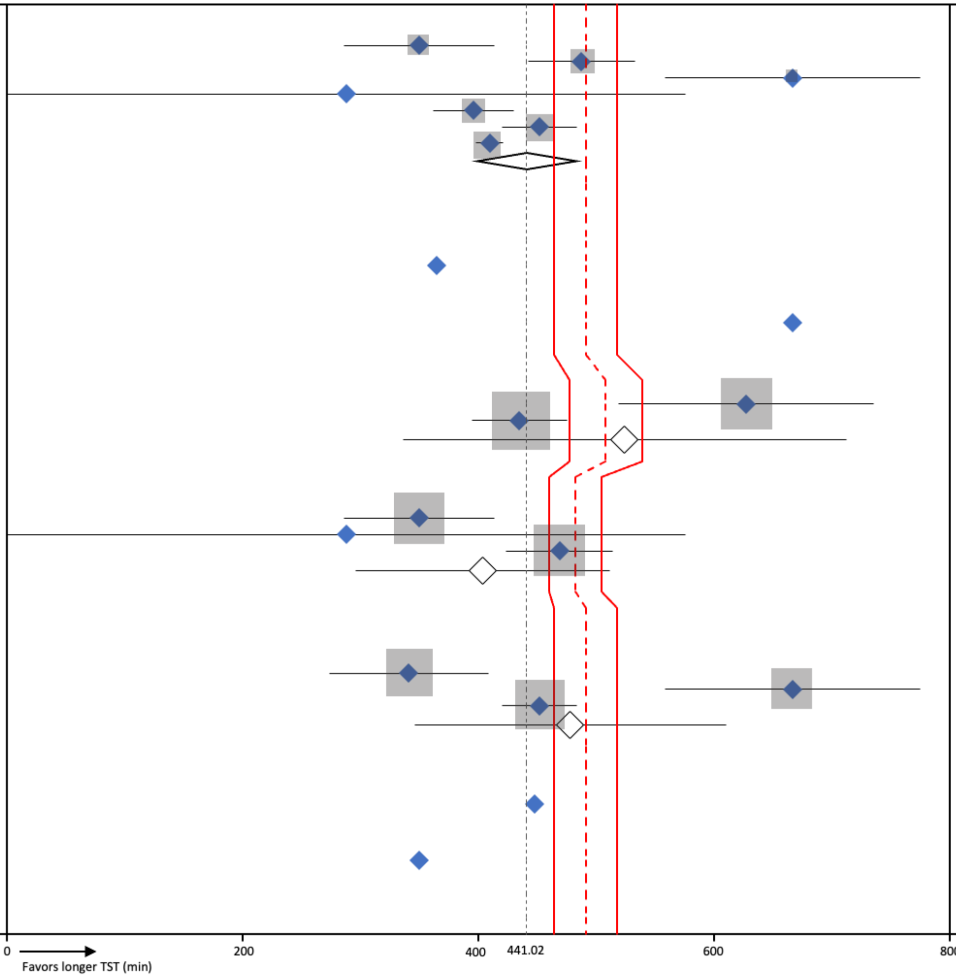

Study or Subgroup	n	Effect size (95% CI)	SD	Z-Value	p-Value	% weight (random)
**Total group**						
Amaddeo et al., (2019)	12	349.75 (285.71, 413.79)	113.20	10.70	< 0.01	14.21
Carotenuto et al., (2013)	13	487.55 (442.00, 533.09)	83.79	20.98	< 0.01	17.06
Hagebeuk et al., (2012b)	4	666.20 (558.01, 774.39)	110.40	12.07	< 0.01	8.74
Schluüter et al., (1995)	2	288.00 (0, 681.95)	284.26	1.43	0.15	1.06
Marcus et al., (1994)	30	396.00 (361.65, 430.35)	96.00	22.59	< 0.01	18.71
Aldrich et al., (1990)	4	451.80 (420.37, 483.23)	32.07	28.18	< 0.01	19.11
Glaze et al., (1986)	11	409.41 (398.15, 420.67)	19.05	71.28	< 0.01	21.12
**Overall**	**76**	**441.02 (399.45, 482.59)**	**184.91**	**20.79**	**< 0.01**	**100**
Heterogeneity: Q (6) = 42.13, I² = 85.76%, p < 0.01 Tau² = 2097.67						
GENE						
MECP2						
Amaddeo et al., (2019)	11	364.91	105.17			
CDKL5						
Hagebeuk et al., (2012b)	4	666.20	110.40			
AGE						
<5 years old						
Hagebeuk et al., (2012b)	3	627.13 (519.16, 735.10)	95.42	11.38	< 0.01	46.48
Aldrich et al., (1990)	2	434.70 (394.32, 475.08)	29.13	21.10	< 0.01	53.52
Over subgroup	5	524.13 (336.02, 712.24)	214.61	5.46	< 0.01	100
Heterogeneity: Q (1) = 10.71, I² = 90.66%, p <0.01 Tau² = 16785.24						
>5 years old						
Amaddeo et al., (2019)	12	349.75 (285.71, 413.79)	113.20	10.70	< 0.01	44.79
Schluüter et al., (1995)	2	288.00 (0, 681.95)	284.26	1.43	0.15	6.59
Aldrich et al., (1990)	2	468.90 (423.62, 514.18)	32.67	20.30	< 0.01	48.62
Over subgroup	16	403.61 (295.59, 511.64)	220.46	7.32	< 0.01	100
Heterogeneity: Q (2) =9.35, I² = 78.62%, p = 0.01 Tau² = 5714.03						
CLINICAL FEATURES						
Epliespy-present						
Amaddeo et al., (2019)	11	340.82 (273.33, 408.30)	114.20	9.90	< 0.01	33.87
Hagebeuk et al., (2012b)	4	666.20 (558.01, 774.39)	110.40	12.07	< 0.01	29.74
Aldrich et al., (1990)	4	451.80 (420.37, 483.23)	32.07	28.18	< 0.01	36.39
Over subgroup	19	477.98 (345.85, 610.11)	239.86	7.09	< 0.01	100
Heterogeneity: Q (2) = 25.37, I² = 92.12%, p <0.01 Tau² = 12233.32						
Epliespy-absent						
Amaddeo et al., (2019)	1	448.00	-			
Scoliosis-present						
Amaddeo et al., (2019)	12	349.75	113.20			
Scoliosis-absent						
No available data						

Blue diamond squares indicate mean with confidence interval of 95% (95% CI) in each study. White diamond squares indicate effect size (i.e., ES, pooled mean) after meta combination, which in the total group the width of the diamond and in the subgroups the error bars represent 95% CI. The size of the grey square indicates the relative weight of the study on the combined ES. The arrows intersect with the Y-axis indicate less than the minimum or more than the maximum of diagrammatic range (0 to 800 minutes). The grey dotted line indicates ES of RTT total group as a reference value for ES in subgroups. The red dashed line indicates the normative average value and red solid lines indicate the range of normative value, which in overall TD population was 490.94 ± 26.56 minutes, in <5 years old TD individuals 507.96 ± 30.81 minutes and in >5 years old TD individuals 482.37 ± 22.08 minutes.

Supplementary 2: Figure 2. Forest plots of sleep macrostructure in RTT — Sleep onset latency (SOL) (Part 1)

Study or Subgroup	n	Effect size (95% CI)	SD	Z-Value	p-Value	% weight (random)
**Total group**						
Sarber et al., (2019)	13	29.67 (14.41, 44.93)	28.08	3.81	< 0.01	14.89
Carotenuto et al., (2013)	13	15.42 (8.82, 22.01)	12.13	4.58	< 0.01	27.88
Hagebeuk et al., (2012b)	4	38.80 (0, 100.73)	63.20	1.23	0.22	1.51
Marcus et al., (1994)	30	48.00 (25.81, 70.19)	62.00	4.24	< 0.01	9.10
Aldrich et al., (1990)	4	8.33 (0, 23.41)	15.39	1.08	0.28	15.10
Glaze et al., (1986)	11	14.01 (9.72, 18.30)	7.26	6.40	< 0.01	31.52
**Overall**	**75**	**19.34 (11.56, 27.13)**	**34.39**	**4.87**	**< 0.01**	**100**
Heterogeneity: Q (5) = 13.41, I^2 = 62.73%, p = 0.02 Tau^2 = 45.25						
GENE						
MECP2						
No available data						
CDKL5						
Hagebeuk et al., (2012b)	4	38.80	63.20			
AGE						
<5 years old						
Hagebeuk et al., (2012b)	3	46.80 (0, 131.61)	74.94	1.08	0.28	10.79
Aldrich et al., (1990)	2	16.35 (0, 45.85)	21.28	1.09	0.28	89.21
Over subgroup	5	19.64 (0, 47.50)	31.79	1.38	0.17	100
Heterogeneity: Q (1) =0.44, I^2 = 0%, p = 0.51 Tau^2 = 0						
>5 years old						
Aldrich et al., (1990)	2	0.30	0.28			
CLINICAL FEATURES						
Epliespy-present						
Hagebeuk et al., (2012b)	4	38.80 (0, 100.73)	63.20	1.23	0.22	5.60
Aldrich et al., (1990)	4	8.33 (0, 23.41)	15.39	1.08	0.28	94.40
Over subgroup	8	10.04 (0, 24.69)	21.15	1.34	0.18	100
Heterogeneity: Q (1) = 0.88, I^2 = 0%, p = 0.35 Tau^2 = 0						
Epliespy-absent						
No available data						
Scoliosis-present						
No available data						
Scoliosis-absent						
No available data						

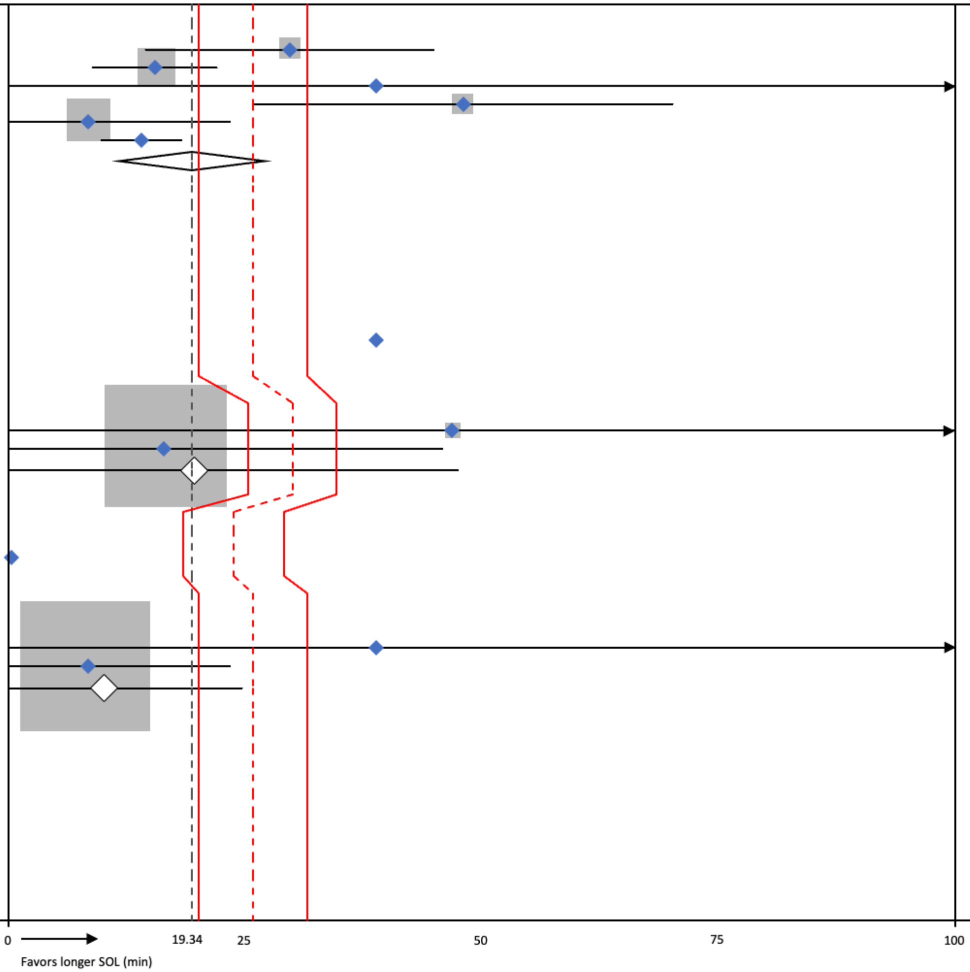

Blue diamond squares indicate mean with confidence interval of 95% (95% CI) in each study. White diamond squares indicate effect size (i.e., ES, pooled mean) after meta combination, which in the total group the width of the diamond and in the subgroups the error bars represent 95% CI. The size of the grey square indicates the relative weight of the study on the combined ES. The arrows intersect with the Y-axis indicate less than the minimum or more than the maximum of diagrammatic range (0 to 100 minutes). The grey dotted line indicates ES of RTT total group as a reference value for ES in subgroups. The red dashed line indicates the normative average value and red solid lines indicate the range of normative value, which in overall TD population was 25.81 ± 5.73 minutes, in <5 years old TD individuals 29.97 ± 4.65 minutes and in >5 years old TD individuals 23.71 ± 5.34minutes.

Supplementary 2: Figure 3. Forest plots of sleep macrostructure in RTT — Wake after sleep onset (WASO) (Part 1)

Study or Subgroup	n	Effect size (95% CI)	SD	Z-Value	p-Value	% weight (random)
**Total group**						
Carotenuto et al., (2013)	13	73.89 (45.18, 102.61)	52.83	5.04	< 0.01	19.96
Hagebeuk et al., (2012b)	4	239.00 (141.98, 336.01)	99.00	4.83	< 0.01	5.35
Schluüter et al., (1995)	2	213.00 (0, 595.19)	275.77	1.09	0.27	0.42
Marcus et al., (1994)	30	51.00 (35.26, 66.74)	44.00	6.35	< 0.01	24.43
Aldrich et al., (1990)	4	26.56 (7.38, 45.74)	19.57	2.71	0.01	23.35
Glaze et al., (1986)	11	71.58 (64.62, 78.55)	11.79	20.14	< 0.01	26.49
**Overall**	**64**	**66.06 (41.00, 91.12)**	**5.17**	**5.17**	**< 0.01**	**100**
Heterogeneity: Q (5) = 35.20, I^2 = 85.79%, p < 0.01 Tau^2 = 604.40						
GENE						
MECP2						
No available data						
CDKL5						
Hagebeuk et al., (2012b)	4	239.00	99.00			
AGE						
<5 years old						
Hagebeuk et al., (2012b)	3	270.07 (163.35, 376.78)	94.31	4.96	< 0.01	47.55
Aldrich et al., (1990)	2	35.38 (3.26, 67.50)	23.18	2.16	0.03	52.45
Over subgroup	5	146.98 (0, 376.70)	262.07	1.25	0.21	100
Heterogeneity: Q (1) =17.04, I^2 = 94.13%, p < 0.01 Tau^2 = 25922.69						
>5 years old						
Schluüter et al., (1995)	2	213.00 (0, 595.19)	275.77	1.09	0.27	0.39
Aldrich et al., (1990)	2	17.74 (0, 41.77)	17.34	1.45	0.15	99.61
Over subgroup	4	18.51 (0, 42.50)	24.48	1.51	0.13	100
Heterogeneity: Q (1) = 1.00, I^2 = 0%, p = 0.32 Tau^2 = 0						
CLINICAL FEATURES						
Epliespy-present						
Hagebeuk et al., (2012b)	4	239.00 (141.98, 336.02)	99.00	4.83	< 0.01	47.39
Aldrich et al., (1990)	4	26.56 (7.38, 45.74)	19.57	2.71	0.01	52.61
Over subgroup	8	127.24 (0, 335.14)	300.03	1.20	0.23	100
Heterogeneity: Q (1) = 17.73, I^2 = 94.36%, p < 0.01 Tau^2 = 21292.38						
Epliespy-absent						
No available data						
Scoliosis-present						
No available data						
Scoliosis-absent						
No available data						

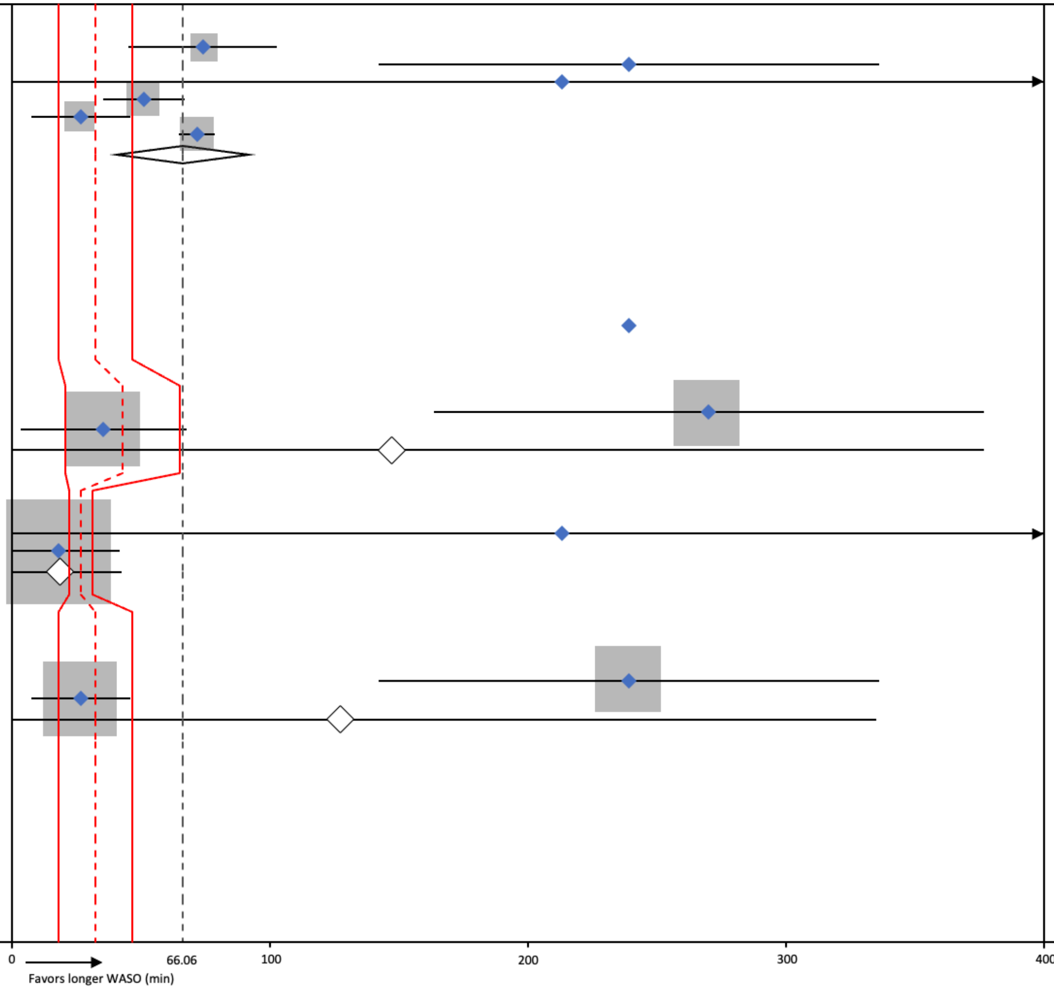

Blue diamond squares indicate mean with confidence interval of 95% (95% CI) in each study. White diamond squares indicate effect size (i.e., ES, pooled mean) after meta combination, which in the total group the width of the diamond and in the subgroups the error bars represent 95% CI. The size of the grey square indicates the relative weight of the study on the combined ES. The arrows intersect with the Y-axis indicate less than the minimum or more than the maximum of diagrammatic range (0 to 400 minutes). The grey dotted line indicates ES of RTT total group as a reference value for ES in subgroups. The red dashed line indicates the normative average value and red solid lines indicate the range of normative value, which in overall TD population was 32.06 ± 14.27 minutes, in <5 years old TD individuals 42.80 ± 22.13 minutes and in >5 years old TD individuals 26.65 ± 4.50 minutes.

Supplementary 2: Figure 4. Forest plots of sleep macrostructure in RTT — Sleep efficiency (SEI) (Part 1)

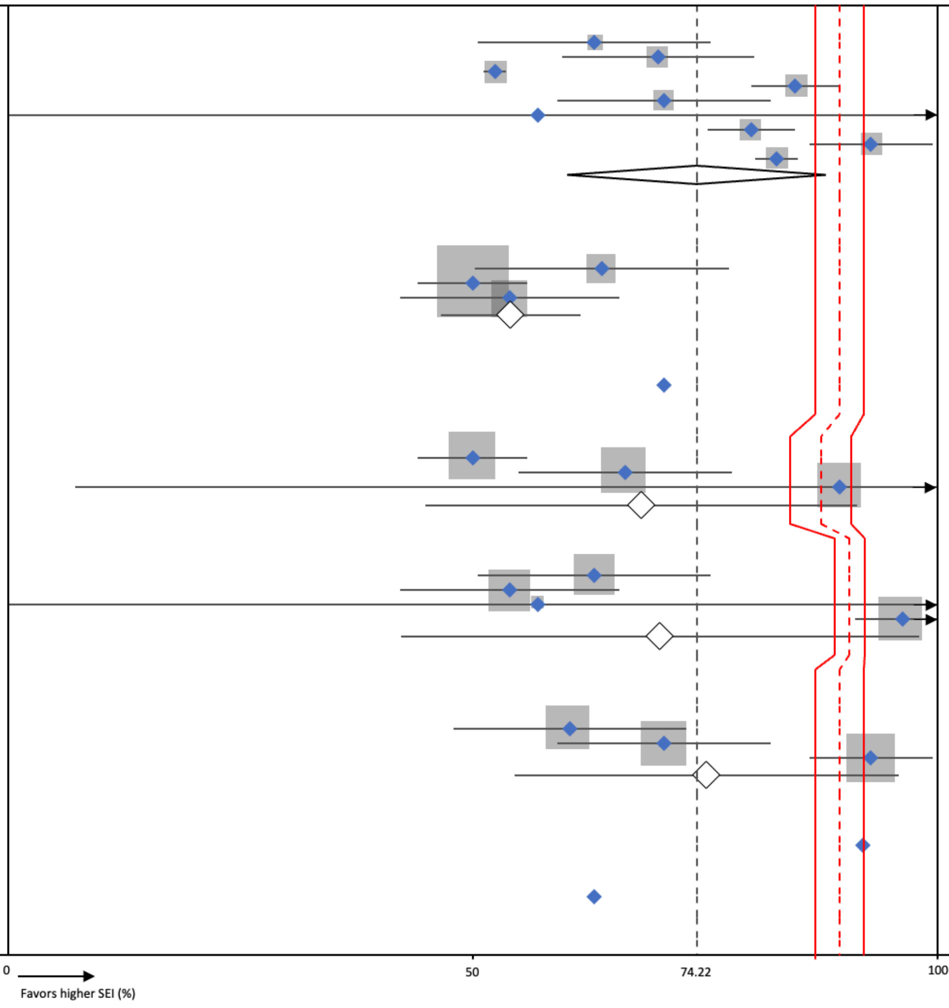

Study or Subgroup	n	Effect size (95% CI)	SD	Z-Value	p-Value	% weight (random)
**Total group**						
Amaddeo et al., (2019)	12	63.08 (50.53, 75.64)	22.19	9.85	< 0.01	11.47
Sarber et al., (2019)	13	70.00 (59.67, 80.33)	19.00	13.28	< 0.01	11.82
Ammanuel et al., (2015)	10	52.40 (51.16, 53.64)	2.00	82.85	< 0.01	12.63
Carotenuto et al., (2013)	13	84.72 (80.00, 89.45)	8.69	35.15	< 0.01	12.47
Hagebeuk et al., (2012b)	4	70.60 (59.13, 82.06)	11.70	12.07	< 0.01	11.65
Schluüter et al., (1995)	2	57.01 (0, 100)	55.77	1.45	0.15	25.77
Marcus et al., (1994)	30	80.00 (75.35, 84.65)	13.00	33.71	< 0.01	12.47
Aldrich et al., (1990)	4	92.87 (86.26, 99.48)	6.74	27.56	< 0.01	12.3
Glaze et al., (1986)	11	82.71 (80.43, 84.98)	3.85	71.28	< 0.01	12.61
**Overall**	**99**	74.22 (60.31, 88.12)	70.58	10.46	< 0.01	100
Heterogeneity: Q (8) = 782.91, I^2 = 98.98%, p < 0.01 Tau^2 = 397.84						
GENE						
MECP2						
Amaddeo et al., (2019)	11	63.91 (50.27, 77.55)	23.08	9.19	< 0.01	21.68
Ammanuel et al., (2015)	4	50.00 (44.12, 55.88)	6.00	16.67	< 0.01	51.79
Ammanuel et al., (2015)	6	54.00(42.24, 65.76)	14.70	9.00	< 0.01	26.53
Overall	21	54.08 (46.56, 61.59)	17.56	14.11	< 0.01	100
Heterogeneity: Q (2) = 3.45, I^2 = 41.96%, p = 0.18 Tau^2 = 19.37						
CDKL5						
Hagebeuk et al., (2012b)	4	70.60	11.70			
AGE						
<5 years old						
Ammanuel et al., (2015)	4	50.00 (44.12, 55.88)	6.00	16.67	< 0.01	34.81
Hagebeuk et al., (2012b)	3	66.43 (55.00, 77.87)	10.11	11.38	< 0.01	32.77
Aldrich et al., (1990)	2	89.50 (7.29, 100)	8.81	14.37	< 0.01	32.41
Over subgroup	9	68.19 (44.98, 91.40)	35.52	5.76	< 0.01	100
Heterogeneity: Q (2) = 34.60, I^2 = 94.22%, p < 0.01 Tau^2 = 393.72						
>5 years old						
Amaddeo et al., (2019)	12	63.08 (50.53, 75.64)	22.19	9.85	< 0.01	29.66
Ammanuel et al., (2015)	6	54.00 (42.24, 65.76)	14.70	9.00	< 0.01	29.88
Schluüter et al., (1995)	2	57.01 (0, 100)	55.77	1.45	0.15	9.21
Aldrich et al., (1990)	2	96.24 (91.21, 100)	3.62	37.55	< 0.01	31.24
Over subgroup	22	70.17 (42.29, 98.05)	66.72	4.93	< 0.01	100
Heterogeneity: Q (3) = 57.70, I^2 =94.80%, p < 0.01 Tau^2 = 641.02						
CLINICAL FEATURES						
Epliespy-present						
Amaddeo et al., (2019)	11	60.45 (47.91, 72.99)	21.22	9.45	< 0.01	32.12
Hagebeuk et al., (2012b)	4	70.60 (59.13, 82.07)	11.70	12.07	< 0.01	32.76
Aldrich et al., (1990)	4	92.87 (86.26, 99.48)	6.74	27.56	< 0.01	35.12
Over subgroup	19	75.16 (54.48, 95.85)	46.01	7.12	< 0.01	100
Heterogeneity: Q (2) = 25.47, I^2 = 92.15%, p < 0.01 Tau^2 = 305.86						
Epliespy-absent						
Amaddeo et al., (2019)	1	92.00	-			
Scoliosis-present						
Amaddeo et al., (2019)	12	63.08	22.19			
Scoliosis-absent						
No available data						

Blue diamond squares indicate mean with confidence interval of 95% (95% CI) in each study. White diamond squares indicate effect size (i.e., ES, pooled mean) after meta combination, which in the total group the width of the diamond and in the subgroups the error bars represent 95% CI. The size of the grey square indicates the relative weight of the study on the combined ES. The arrows intersect with the Y-axis indicate less than the minimum or more than the maximum of diagrammatic range (0 to 100 %). The grey dotted line indicates ES of RTT total group as a reference value for ES in subgroups. The red dashed line indicates the normative average value and red solid lines indicate the range of normative value, which in overall TD population was 89.53 ± 2.59 %, in <5 years old TD individuals 87.50 ± 3.30 % and in >5 years old TD individuals 90.56 ± 1.61 %.

Supplementary 2: Figure 5. Forest plots of sleep macrostructure in RTT — Non-rapid eye movement sleep stage 1 (Stage N1) (Part 1)

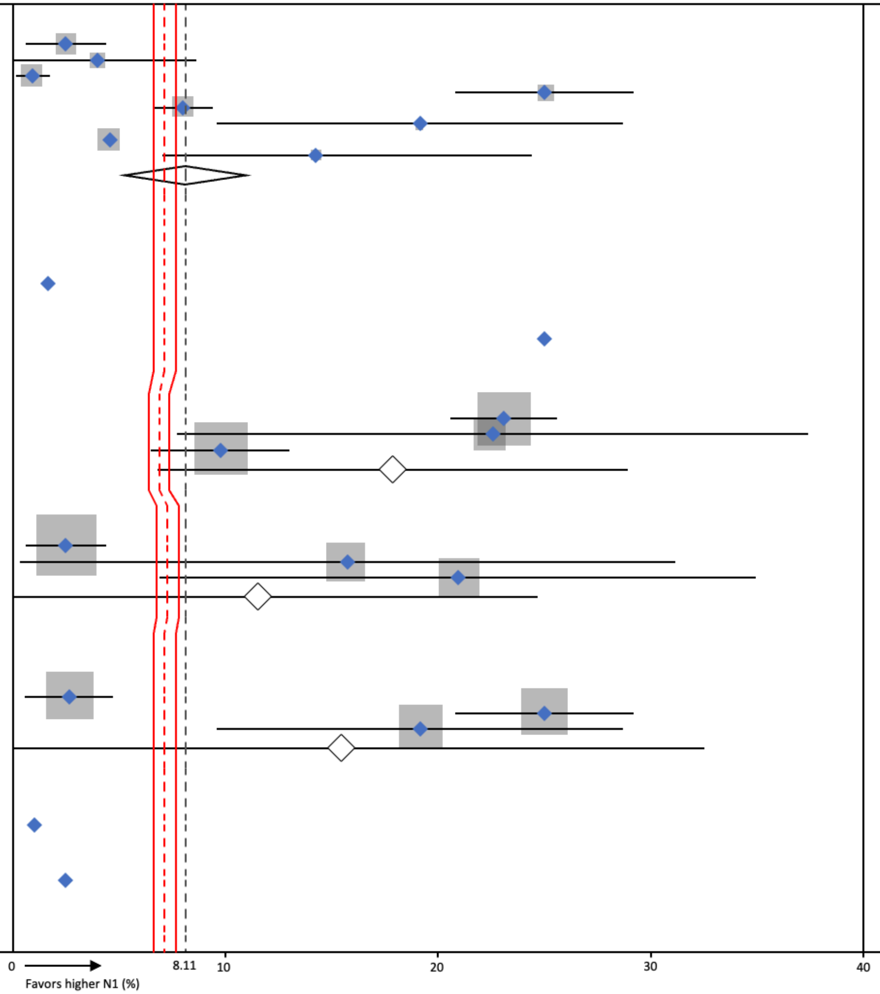

Study or Subgroup	n	Effect size (95% CI)	SD	Z-Value	p-Value	% weight (random)
**Total group**						
Amaddeo et al., (2019)	12	2.50 (0.59, 4.41)	3.37	2.57	0.01	15.26
Sarber et al., (2019)	13	4.00 (0, 8.65)	8.55	1.69	0.09	11.18
Carotenuto et al., (2013)	13	0.95 (0.17, 1.74)	1.45	2.38	0.02	16.26
Hagebeuk et al., (2012b)	4	25.00 (20.79, 29.21)	4.30	11.63	< 0.01	11.85
Marcus et al., (1994)	30	8.00 (6.57, 9.43)	4.00	10.95	< 0.01	15.77
Aldrich et al., (1990)	4	19.15 (9.60, 28.70)	9.74	3.93	< 0.01	5.49
Glaze et al., (1986)	11	4.57 (4.32, 4.83)	0.43	35.03	< 0.01	16.46
Nomura et al., (1985)	5	14.23 (7.04, 21.42)	8.19	3.88	< 0.01	7.72
**Overall**	**92**	**8.11 (5.37, 10.85)**	**13.4**	**5.81**	**< 0.01**	**100**
Heterogeneity: Q (7) = 211.67, I² = 96.69%, p < 0.01 Tau² = 11.84						
GENE						
MECP2						
Amaddeo et al., (2019)	12	1.64	1.63			
CDKL5						
Hagebeuk et al., (2012b)	4	25.00	4.3			
AGE						
<5 years old						
Hagebeuk et al., (2012b)	3	23.08 (20.57, 25.60)	2.23	17.95	< 0.01	38.72
Aldrich et al., (1990)	2	22.56 (7.70, 37.41)	10.72	2.98	< 0.01	23.06
Nomura et al., (1985)	3	9.76 (6.51, 13.00)	2.89	5.90	< 0.01	38.22
Over subgroup	8	17.87 (6.81, 28.92)	15.95	3.17	< 0.01	100
Heterogeneity: Q (2) = 40.79, I² = 95.10%, p < 0.01 Tau² = 80.48						
>5 years old						
Amaddeo et al., (2019)	12	2.50 (0.59, 4.41)	3.37	2.57	0.01	43.38
Aldrich et al., (1990)	2	15.75 (0.34, 31.15)	11.12	2.00	0.05	27.37
Nomura et al., (1985)	2	20.93 (6.91, 34.95)	10.11	2.93	< 0.01	29.25
Over subgroup	16	11.52 (0, 24.68)	26.86	1.72	0.09	100
Heterogeneity: Q (2) = 9.18, I² = 78.21%, p = 0.01 Tau² = 102.99						
CLINICAL FEATURES						
Epliespy-present						
Amaddeo et al., (2019)	11	2.64 (0.57, 4.71)	3.50	2.50	0.01	34.59
Hagebeuk et al., (2012b)	4	25 (20.79, 29.21)	4.30	11.63	< 0.01	34.05
Aldrich et al., (1990)	4	19.15 (9.60, 28.70)	9.74	3.93	< 0.01	31.36
Over subgroup	19	15.43 (0, 32.52)	38.00	1.77	0.08	100
Heterogeneity: Q (2) = 93.20, I² = 97.85%, p < 0.01 Tau² = 218.61						
Epliespy-absent						
Amaddeo et al., (2019)	1	1.00	-			
Scoliosis-present						
Amaddeo et al., (2019)	12	2.50	3.37			
Scoliosis-absent						
No available data						

Blue diamond squares indicate mean with confidence interval of 95% (95% CI) in each study. White diamond squares indicate effect size (i.e., ES, pooled mean) after meta combination, which in the total group the width of the diamond and in the subgroups the error bars represent 95% CI. The size of the grey square indicates the relative weight of the study on the combined ES. The arrows intersect with the Y-axis indicate less than the minimum or more than the maximum of diagrammatic range (0 to 40 %). The grey dotted line indicates ES of RTT total group as a reference value for ES in subgroups. The red dashed line indicates the normative average value and red solid lines indicate the range of normative value, which in overall TD population was 7.15 ± 0.52 %, in <5 years old TD individuals 6.88 ± 0.47 % and in >5 years old TD individuals 7.28 ± 0.54 %.

Supplementary 2: Figure 6. Forest plots of sleep macrostructure in RTT — Non-rapid eye movement sleep stage 2 (Stage N2) (Part 1)

Study or Subgroup	n	Effect size (95% CI)	SD	Z-Value	p-Value	% weight (random)
**Total group**						
Amaddeo et al., (2019)	12	32.17 (19.98, 44.35)	21.54	5.17	< 0.01	11.20
Sarber et al., (2019)	13	44.00 (13.02, 74.98)	57	2.78	0.01	4.24
Carotenuto et al., (2013)	13	33.86 (28.66, 39.07)	9.57	12.76	< 0.01	14.85
Hagebeuk et al., (2012b)	4	43.30 (28.70, 57.90)	14.9	5.81	< 0.01	9.90
Marcus et al., (1994)	30	55.00 (51.06, 58.94)	11	27.39	< 0.01	15.33
Aldrich et al., (1990)	4	36.17 (29.46, 42.88)	6.85	10.56	< 0.01	14.17
Glaze et al., (1986)	11	49.85 (49.42, 50.27)	0.72	229.11	< 0.01	16.01
Nomura et al., (1985)	5	26.88 (20.64, 33.30)	7.32	8.21	< 0.01	14.31
**Overall**	**92**	**40.61 (32.72, 47.60)**	**36.4**	**10.58**	**< 0.01**	**100**
Heterogeneity: Q (7) = 115.84, I^2 = 93.96%, p < 0.01 Tau^2 = 89.92						
GENE						
MECP2						
Amaddeo et al., (2019)	11	27.73	15.82			
CDKL5						
Hagebeuk et al., (2012b)	4	43.30	14.9			
AGE						
<5 years old						
Hagebeuk et al., (2012b)	3	44.36 (23.92, 64.80)	18.06	4.25	< 0.01	6.23
Aldrich et al., (1990)	2	31.43 (21.74, 41.13)	6.99	6.36	< 0.01	24.20
Nomura et al., (1985)	3	28.43 (23.88, 32.99)	4.02	14.35	< 0.01	69.57
Over subgroup	8	30.15 (24.94, 35.37)	7.52	11.34	< 0.01	100
Heterogeneity: Q (2) = 2.39, I^2 = 16.37%, p = 0.30 Tau^2 = 4.77						
>5 years old						
Amaddeo et al., (2019)	12	32.17 (19.98, 44.35)	21.54	5.17	< 0.01	28.30
Aldrich et al., (1990)	2	40.90 (38.83, 42.98)	1.50	38.58	< 0.01	53.34
Nomura et al., (1985)	2	24.54 (6.79, 42.29)	12.81	2.71	0.01	18.36
Over subgroup	16	35.43 (26.10, 44.75)	19.03	7.45	< 0.01	100
Heterogeneity: Q (2) = 5.04, I^2 = 60.35%, p = 0.08 Tau^2 = 41.32						
CLINICAL FEATURES						
Epliespy-present						
Amaddeo et al., (2019)	11	32.00 (18.65, 45.35)	22.58	4.70	< 0.01	17.28
Hagebeuk et al., (2012b)	4	43.30 (28.70, 57.90)	14.90	5.81	< 0.01	14.43
Aldrich et al., (1990)	4	36.17 (29.46, 42.88)	6.85	10.56	< 0.01	68.29
Over subgroup	19	36.48 (30.93, 42.03)	12.34	12.89	< 0.01	100
Heterogeneity: Q (2) = 1.28, I^2 = 0%, p = 0.53 Tau^2 = 0						
Epliespy-absent						
Amaddeo et al., (2019)	1	34.00	-			
Scoliosis-present						
Amaddeo et al., (2019)	12	32.17	21.54			
Scoliosis-absent						
No available data						

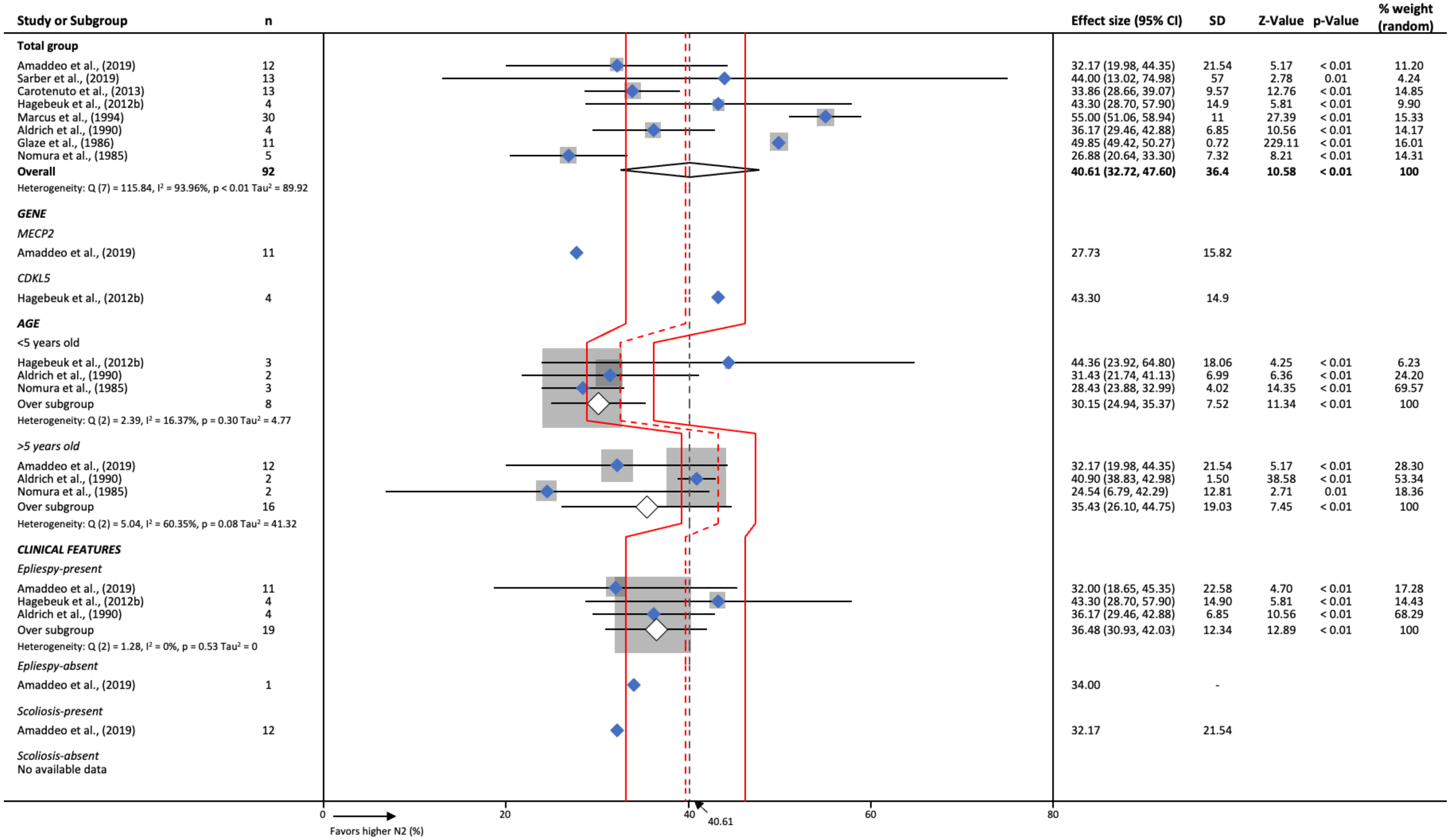

Blue diamond squares indicate mean with confidence interval of 95% (95% CI) in each study. White diamond squares indicate effect size (i.e., ES, pooled mean) after meta combination, which in the total group the width of the diamond and in the subgroups the error bars represent 95% CI. The size of the grey square indicates the relative weight of the study on the combined ES. The arrows intersect with the Y-axis indicate less than the minimum or more than the maximum of diagrammatic range (0 to 80 %). The grey dotted line indicates ES of RTT total group as a reference value for ES in subgroups. The red dashed line indicates the normative average value and red solid lines indicate the range of normative value, which in overall TD population was 39.69 ± 6.56 %, in <5 years old TD individuals 32.54 ± 3.69 % and in >5 years old TD individuals 42.30 ± 4.08 %.

Supplementary 2: Figure 7. Forest plots of sleep macrostructure in RTT — Non-rapid eye movement sleep stage 3 (Stage N3) (Part 1)

Study or Subgroup	n	Effect size (95% CI)	SD	Z-Value	p-Value	% weight (random)
**Total group**						
Amaddeo et al., (2019)	12	52.50 (38.28, 66.73)	25.14	7.23	< 0.01	7.42
Sarber et al., (2019)	13	32.00 (0.88, 63.12)	57.25	2.02	0.04	2.84
Ammanuel et al., (2015)	10	52.00 (46.12, 57.88)	9.49	17.33	< 0.01	11.48
Carotenuto et al., (2013)	13	48.66 (40.42, 56.91)	15.17	11.56	< 0.01	10.35
Hagebeuk et al., (2012b)	4	22.80 (9.96, 35.64)	13.10	3.48	< 0.01	8.06
Marcus et al., (1994)	30	18.00 (14.78, 21.23)	9.00	10.95	< 0.01	12.46
Aldrich et al., (1990)	4	27.37 (21.72, 33.02)	5.77	9.49	< 0.01	11.57
Segawa et al., (1990)	12	35.57 (31.99, 39.15)	6.33	19.47	< 0.01	12.35
Glaze et al., (1986)	11	33.83 (33.46, 34.20)	0.63	179.40	< 0.01	12.92
Nomura et al., (1985)	5	34.62 (36.77, 42.47)	8.96	8.64	< 0.01	10.55
**Overall**	**114**	**35.47 (29.53, 41.41)**	**32.35**	**11.71**	**< 0.01**	**100**
Heterogeneity: Q (9) = 156.99, I^2 = 94.27%, p < 0.01 Tau^2 = 71.00						
GENE						
MECP2						
Amaddeo et al., (2019)	11	57.27 (45.53, 69.01)	19.86	9.56	< 0.01	20.06
Ammanuel et al., (2015)	10	52.00 (46.12, 57.88)	9.49	17.33	< 0.01	79.94
Over subgroup	21	53.06 (47.80, 58.31)	12.29	19.78	< 0.01	100
Heterogeneity: Q (1) =0.62, I^2 = 0%, p = 0.43 Tau^2 = 0						
CDKL5						
Hagebeuk et al., (2012b)	4	22.80	13.1			
AGE						
<5 years old						
Hagebeuk et al., (2012b)	3	25.36 (8.62, 42.11)	14.80	2.97	< 0.01	18.14
Segawa et al., (1990)	6	29.24 (17.54, 40.94)	14.62	4.90	< 0.01	23.04
Aldrich et al., (1990)	2	22.80 (19.81, 25.79)	2.16	14.93	< 0.01	30.32
Nomura et al., (1985)	3	40.55 (34.65, 46.45)	5.21	13.47	< 0.01	28.50
Over subgroup	14	29.81 (18.74, 40.88)	21.14	5.28	< 0.01	100
Heterogeneity: Q (3) = 27.88, I^2 = 89.24%, p < 0.01 Tau^2 = 102.91						
>5 years old						
Amaddeo et al., (2019)	12	52.50 (38.28, 66.72)	25.14	7.23	< 0.01	17.12
Segawa et al., (1990)	6	41.90 (33.98, 49.82)	9.94	10.37	< 0.01	24.64
Aldrich et al., (1990)	2	31.93 (27.13, 36.73)	3.46	13.04	< 0.01	28.16
Nomura et al., (1985)	2	25.72 (23.41, 28.04)	1.67	21.78	< 0.01	30.07
Over subgroup	22	36.04 (27.19, 44.89)	21.18	7.98	< 0.01	100
Heterogeneity: Q (3) = 29.40, I^2 = 89.80%, p < 0.01 Tau^2 = 66.42						
CLINICAL FEATURES						
Epilepsy-present						
Amaddeo et al., (2019)	11	32.00 (18.65, 45.35)	13.10	4.70	< 0.01	13.07
Hagebeuk et al., (2012b)	4	22.80 (9.96, 35.64)	22.58	3.48	< 0.01	14.12
Aldrich et al., (1990)	4	27.37 (21.72, 33.02)	5.77	9.49	< 0.01	72.81
Over subgroup	19	27.33 (22.5, 32.15)	10.73	11.10	< 0.01	100
Heterogeneity: Q (2) = 0.95, I^2 = 0%, p = 0.62 Tau^2 = 0						
Epilespy-absent						
Amaddeo et al., (2019)	1	37.00	-			
Scoliosis-present						
Amaddeo et al., (2019)	12	52.50	25.14			
Scoliosis-absent						
No available data						

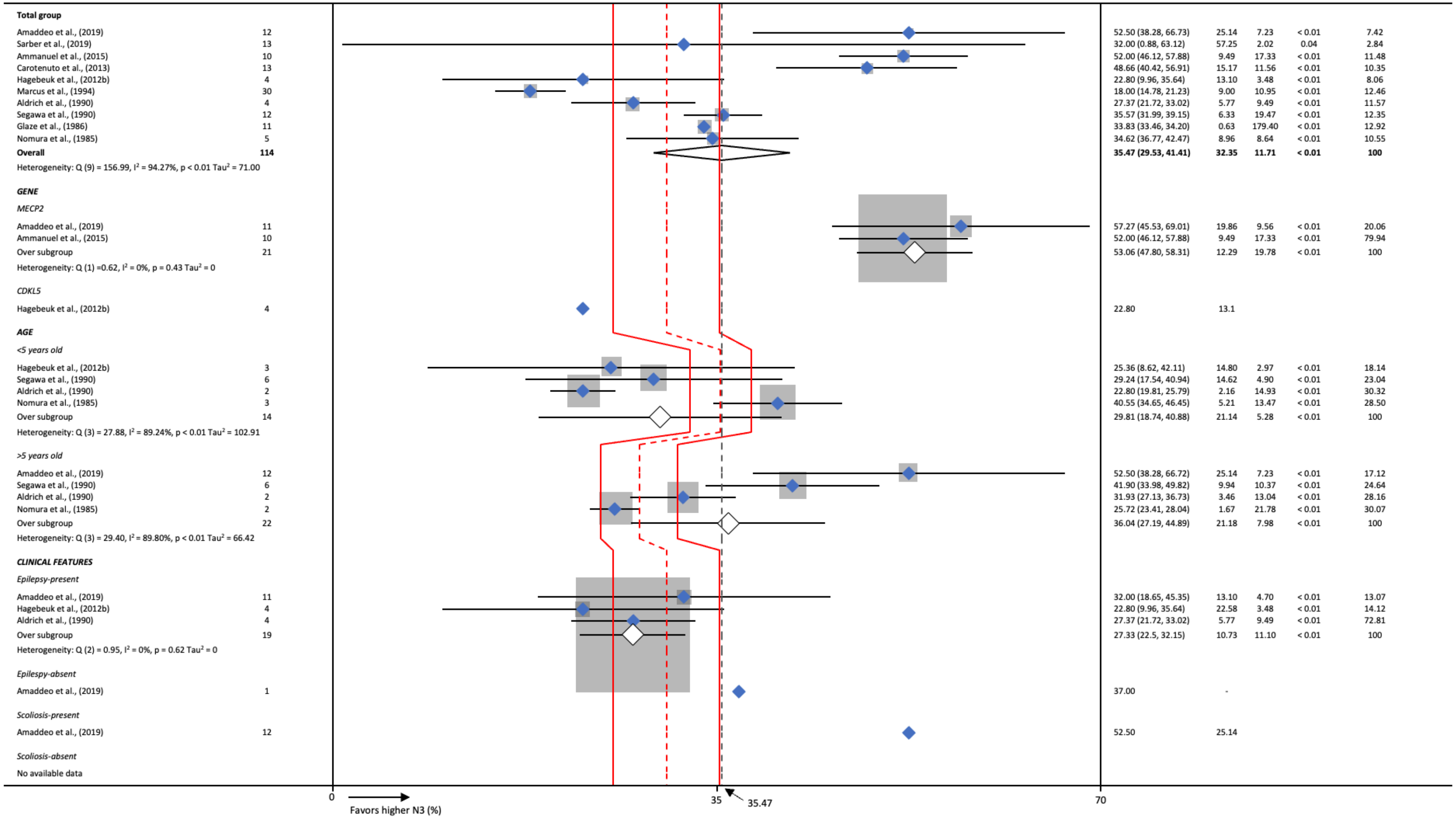

Blue diamond squares indicate mean with confidence interval of 95% (95% CI) in each study. White diamond squares indicate effect size (i.e., ES, pooled mean) after meta combination, which in the total group the width of the diamond and in the subgroups the error bars represent 95% CI. The size of the grey square indicates the relative weight of the study on the combined ES. The arrows intersect with the Y-axis indicate less than the minimum or more than the maximum of diagrammatic range (0 to 70 %). The grey dotted line indicates ES of RTT total group as a reference value for ES in subgroups. The red dashed line indicates the normative average value and red solid lines indicate the range of normative value, which in overall TD population was 30.40 ± 4.84 %, in <5 years old TD individuals 35.34 ± 2.81 % and in >5 years old TD individuals 27.92 ± 3.49 %.

Supplementary 2: Figure 8. Forest plots of sleep macrostructure in RTT — Rapid eye movement sleep (REM) (Part 1)

Study or Subgroup	n	Effect size (95% CI)	SD	Z-Value	p-Value	% weight (random)
**Total group**						
Amaddeo et al., (2019)	12	12.17 (6.28, 18.05)	10.40	4.05	< 0.01	11.10
Sarber et al., (2019)	13	17.00 (0, 35.48)	34.00	1.80	0.07	3.85
Carotenuto et al., (2013)	13	13.82 (9.24, 18.41)	8.44	5.91	< 0.01	12.11
Hagebeuk et al., (2012b)	4	8.90 (2.43, 15.37)	6.60	2.70	0.01	10.63
Marcus et al., (1994)	30	19.00 (16.14, 21.86)	8.00	13.01	< 0.01	13.25
Aldrich et al., (1990)	4	17.13 (9.56, 24.70)	7.72	4.44	< 0.01	9.75
Segawa et al., (1990)	12	21.47 (21.15, 21.78)	0.57	131.61	< 0.01	14.08
Glaze et al., (1986)	11	13.05 (12.54, 13.57)	0.87	50.00	< 0.01	14.06
Nomura et al., (1985)	5	24.28 (18.46, 30.09)	6.63	8.64	< 0.01	11.16
**Overall**	**104**	**16.38 (12.12, 20.64)**	**22.15**	**7.82**	**< 0.01**	**100**
Heterogeneity: Q (8) = 769.71, I^2 = 98.96%, p < 0.01 Tau^2 = 33.47						
GENE						
MECP2						
Amaddeo et al., (2019)	11	12.64	10.77			
CDKL5						
Hagebeuk et al., (2012b)	4	8.90	6.6			
AGE						
<5 years old						
Hagebeuk et al., (2012b)	3	7.20 (0, 15.05)	6.94	1.80	0.07	19.99
Segawa et al., (1990)	6	22.03 (17.35, 26.71)	5.85	9.23	< 0.01	27.12
Aldrich et al., (1990)	2	23.05 (21.00, 25.11)	1.48	21.97	< 0.01	32.23
Nomura et al., (1985)	3	21.26 (13.72, 28.80)	6.66	5.53	< 0.01	20.65
Over subgroup	14	19.24 (13.74, 24.73)	10.50	6.86	< 0.01	100
Heterogeneity: Q (3) = 14.69, I^2 = 79.58%, p < 0.01 Tau^2 = 23.31						
>5 years old						
Amaddeo et al., (2019)	12	12.17 (6.28, 18.05)	10.40	4.05	< 0.01	25.56
Segawa et al., (1990)	6	20.90 (16.10, 25.70)	6.04	8.53	< 0.01	26.90
Aldrich et al., (1990)	2	11.21 (2.85, 19.56)	6.03	2.63	0.01	22.20
Nomura et al., (1985)	2	28.81 (22.76, 34.85)	4.36	9.34	< 0.01	25.35
Over subgroup	22	18.52 (10.81, 26.23)	18.45	4.71	< 0.01	100.0
Heterogeneity: Q (3) = 19.17, I^2 = 84.35%, p < 0.01 Tau^2 = 51.50						
CLINICAL FEATURES						
Epliespy-present						
Amaddeo et al., (2019)	4	8.90 (2.43, 15.37)	9.74	2.70	0.01	33.72
Hagebeuk et al., (2012b)	4	17.13 (9.56, 24.70)	6.60	4.44	< 0.01	26.72
Aldrich et al., (1990)	11	10.82 (5.06, 16.58)	7.72	3.68	< 0.01	39.56
Over subgroup	19	11.86 (7.41, 16.31)	9.90	5.22	< 0.01	100
Heterogeneity: Q (2) = 2.79, I^2 = 28.28%, p = 0.25 Tau^2 = 4.41						
Epliespy-absent						
Amaddeo et al., (2019)	1	27.00	-			
Scoliosis-present						
Amaddeo et al., (2019)	12	12.17	10.40			
Scoliosis-absent						
No available data						

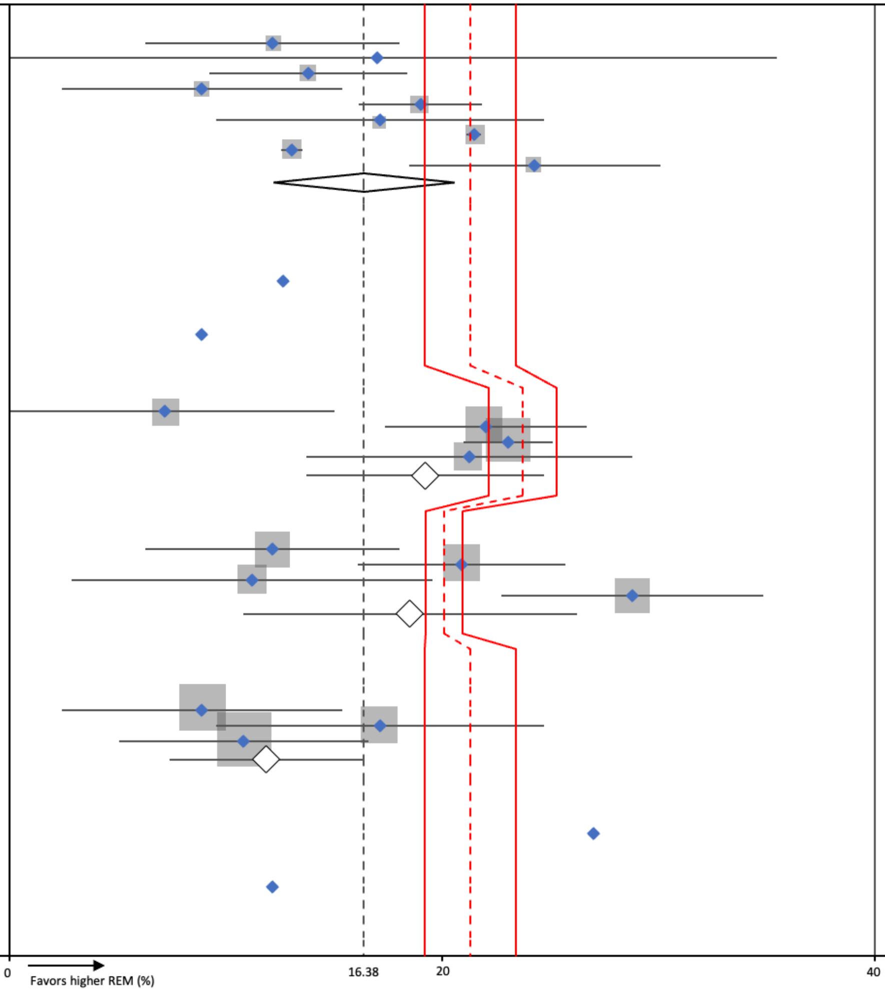

Blue diamond squares indicate mean with confidence interval of 95% (95% CI) in each study. White diamond squares indicate effect size (i.e., ES, pooled mean) after meta combination, which in the total group the width of the diamond and in the subgroups the error bars represent 95% CI. The size of the grey square indicates the relative weight of the study on the combined ES. The arrows intersect with the Y-axis indicate less than the minimum or more more than the maximum of diagrammatic range (0 to 40 %). The gray dotted line indicates ES of RTT total group as a reference value for ES in subgroups. The red dashed line indicates the normative average value and red solid lines indicate the range of normative value, which in overall TD population was 21.32 ± 2.10 %, in <5 years old TD individuals 23.74 ± 1.54 % and in >5 years old TD individuals 20.11 ± 0.84 %.

Supplementary 2: Figure 9. Forest plots of sleep respiratory in RTT — Apnea hypopnea index (AHI) (Part 1)

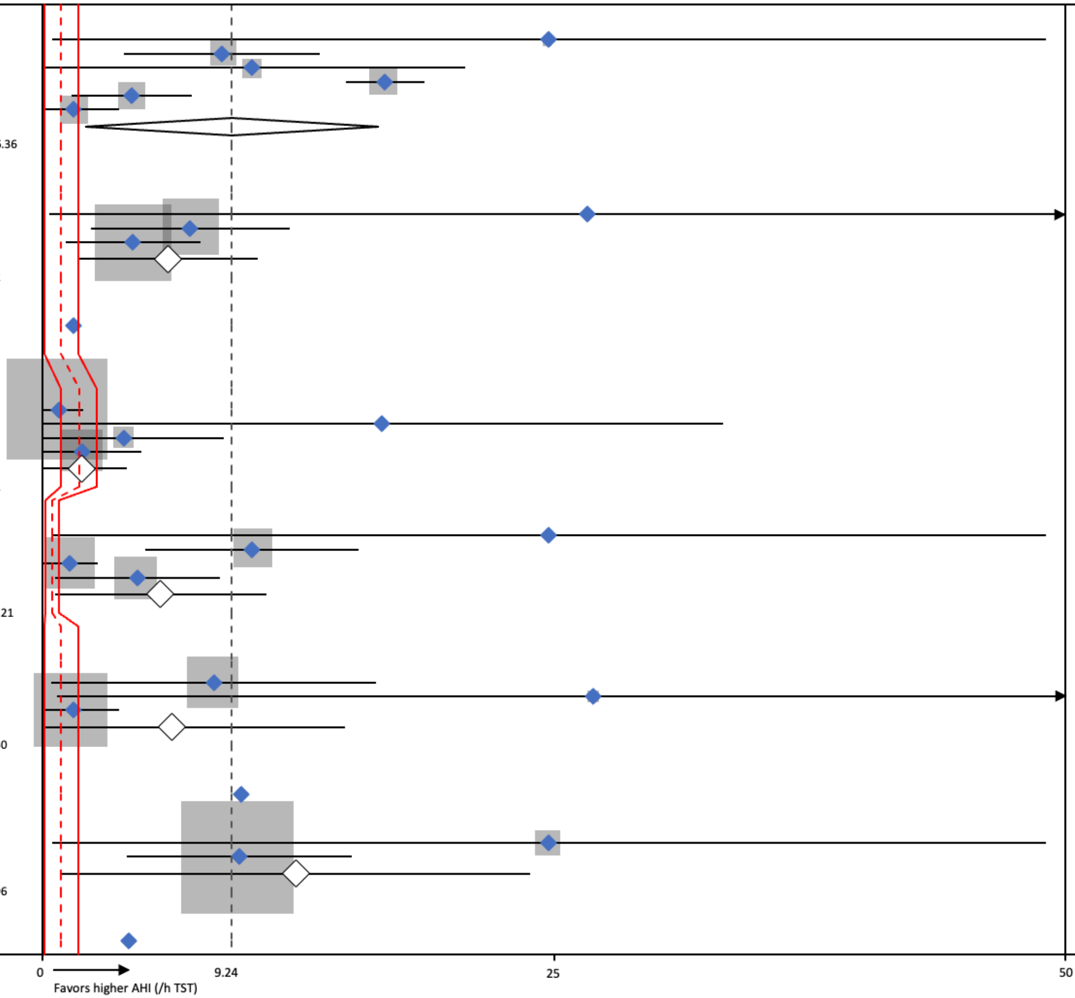

Study or Subgroup	n	Effect size (95% CI)	SD	Z-Value	p-Value	% weight (random)
**Total group**						
Amaddeo et al., (2019)	12	24.75 (0.45, 49.05)	42.95	2.00	0.05	6.21
Sarber et al., (2019)	13	8.77 (3.98, 13.56)	8.82	3.59	< 0.01	18.90
Bassett et al., (2016)	12	10.22 (0, 20.64)	18.41	1.92	0.05	14.45
Carotenuto et al., (2013)	13	16.74 (14.83, 18.65)	3.52	17.17	< 0.01	20.31
Hagebeuk et al., (2012 a)	10	4.36 (1.42, 7.30)	4.74	2.91	< 0.01	19.93
Hagebeuk et al., (2012b)	4	1.48 (0, 3.72)	2.29	1.29	0.20	20.20
**Overall**	**64**	**9.24 (1.99, 16.48)**	**29.60**	**2.50**	**0.01**	**100**
Heterogeneity: Q (5) =117.34, I^2 = 95.74%, p < 0.01 Tau^2 = 66.36						
GENE						
MECP2						
Amaddeo et al., (2019)	11	26.64 (0.33, 52.95)	44.52	1.98	0.05	2.65
Sarber et al., (2019)	11	7.20 (2.34, 12.06)	8.23	2.90	< 0.01	40.82
Hagebeuk et al., (2012 a)	9	4.41 (1.13, 7.69)	5.02	2.64	0.01	56.53
Over subgroup	31	6.14 (1.79, 10.49)	12.36	2.76	0.01	100
Heterogeneity: Q (2) = 3.37, I^2 = 40.71%, p = 0.19 Tau^2 = 5.92						
CDKL5						
Hagebeuk et al., (2012b)	4	1.48	2.29			
AGE						
<5 years old						
Sarber et al., (2019)	2	0.80 (0, 1.98)	0.85	1.33	0.18	53.12
Bassett et al., (2016)	7	16.57 (0, 33.25)	22.51	1.95	0.05	1.69
Hagebeuk et al., (2012 a)	4	3.95 (0, 8.85)	5.00	1.58	0.11	15.26
Hagebeuk et al., (2012b)	3	1.93 (0, 4.84)	2.57	1.30	0.19	29.92
Over subgroup	16	1.89 (0, 4.09)	4.48	1.68	0.09	100
Heterogeneity: Q (3) = 5.16, I^2 = 41.82%, p = 0.16 Tau^2 = 2.01						
>5 years old						
Amaddeo et al., (2019)	12		42.95	2.00	0.05	4.05
Sarber et al., (2019)	11	10.22 (4.99, 15.44)	8.84	3.83	< 0.01	27.53
Bassett et al., (2016)	5	1.32 (0, 2.67)	1.54	1.92	0.06	37.31
Hagebeuk et al., (2012 a)	6	4.63 (0.60, 8.65)	5.02	2.26	0.02	31.11
Over subgroup	34	5.75 (0.58, 10.92)	15.39	2.18	0.03	100
Heterogeneity: Q (3) = 15.40, I^2 = 80.52%, p < 0.01 Tau^2 = 18.21						
CLINICAL FEATURES						
Epliespy-present						
Sarber et al., (2019)	7	8.36 (0.40, 16.31)	10.74	2.06	0.04	37.44
Amaddeo et al., (2019)	11	26.91 (0.70, 53.12)	44.36	2.01	0.04	8.81
Hagebeuk et al., (2012b)	4	1.48 (0, 3.72)	2.29	1.29	0.20	53.75
Over subgroup	22	6.29 (0, 14.77)	20.29	1.45	0.15	100
Heterogeneity: Q (2) = 6.11, I^2 = 67.28%, p = 0.05 Tau^2 = 33.50						
Epliespy-absent						
Sarber et al., (2019)	5	9.70	7.61			
Scoliosis-present						
Amaddeo et al., (2019)	12	24.75 (0.45, 49.05)	42.95	2.00	0.05	18.23
Sarber et al., (2019)	11	9.60 (4.10, 15.10)	9.31	3.42	< 0.01	81.77
Over subgroup	23	12.36 (0.90, 23.83)	28.05	2.11	0.03	100
Heterogeneity: Q (1) = 1.42, I^2 = 29.59%, p = 0.23 Tau^2 = 33.96						
Scoliosis-absent						
Sarber et al., (2019)	2	4.20	3.96			

Blue diamond squares indicate mean with confidence interval of 95% (95% CI) in each study. White diamond squares indicate effect size (i.e., ES, pooled mean) after meta combination, which in the total group the width of the diamond and in the subgroups the error bars represent 95% CI. The size of the grey square indicates the relative weight of the study on the combined ES. The arrows intersect with the Y-axis indicate less than the minimum or more than the maximum of diagrammatic range (0 to 50). The grey dotted line indicates ES of RTT total group as a reference value for ES in subgroups. The red dashed line indicates the normative average value and red solid lines indicate the range of normative value, which in overall TD population was 0.89 ± 0.84 /h TST, in <5 years old TD individuals 1.77 ± 0.88 /h TST and in >5 years old TD individuals 0.45 ± 0.32 /h TST.

Supplementary 2: Figure 10. Forest plots of sleep respiratory in RTT — Obstructive Apnea hypopnea index (OAHI) (Part 1)

Study or Subgroup	n	Effect size (95% CI)	SD	Z-Value	p-Value	% weight (random)
**Total group**						
Amaddeo et al., (2019)	12	10.50 (3.14, 17.86)	13.01	2.80	0.01	18.86
Sarber et al., (2019)	13	6.51 (2.75, 10.27)	6.91	3.40	< 0.01	37.09
Hagebeuk et al., (2012 a)	10	2.73 (0, 5.48)	4.44	1.94	0.05	44.04
**Overall**	**25**	**5.60 (1.68, 9.52)**	**11.83**	**2.80**	**0.01**	**100**
Heterogeneity: Q (2) = 5.24, I^2 = 61.80%, p = 0.07 Tau^2 = 7.12						
GENE						
MECP2						
Amaddeo et al., (2019)	11	11.09 (3.13, 19.05)	13.47	2.73	0.01	14.84
Sarber et al., (2019)	11	5.16 (1.37, 8.95)	6.41	2.67	0.01	38.65
Hagebeuk et al., (2012 a)	9	2.96 (0, 5.99)	4.64	1.91	0.06	46.51
Over subgroup	31	5.02 (1.58, 8.45)	9.76	2.86	< 0.01	100
Heterogeneity: Q (2) = 3.37, I^2 = 46.45%, p = 0.15 Tau^2 = 4.22						
CDKL5						
No available data						
AGE						
<5 years old						
Sarber et al., (2019)	2	0.45 (0, 1.34)	0.64	0.99	0.32	87.15
Hagebeuk et al., (2012 a)	4	3.23 (0, 8.00)	4.87	1.32	0.19	12.85
Over subgroup	6	0.81 (0, 2.63)	2.28	0.87	0.39	100
Heterogeneity: Q (1) = 1.26, I^2 = 20.39%, p = 0.26 Tau^2 = 0.79						
>5 years old						
Amaddeo et al., (2019)	12	10.50 (3.14, 17.86)	13.01	2.80	0.01	22.52
Sarber et al., (2019)	11	7.61 (3.49, 11.73)	6.97	3.62	< 0.01	37.44
Hagebeuk et al., (2012 a)	6	2.40 (0, 6.06)	4.57	1.29	0.20	40.03
Over subgroup	29	6.18 (1.59, 10.76)	12.60	2.64	0.01	100
Heterogeneity: Q (2) = 5.54, I^2 = 63.87%, p = 0.06 Tau^2 = 10.19						
CLINICAL FEATURES						
Epliespy-present						
Amaddeo et al., (2019)	11	11.45 (3.66, 19.25)	13.19	2.88	< 0.01	36.39
Sarber et al., (2019)	8	5.29 (0.70, 9.87)	6.62	2.26	0.02	63.61
Over subgroup	19	7.53 (1.72, 13.35)	12.93	2.54	0.01	100
Heterogeneity: Q (1) = 1.79, I^2 = 44.00%, p = 0.18 Tau^2 = 8.37						
Epliespy-absent						
Sarber et al., (2019)	5	8.46	7.68			
Scoliosis-present						
Amaddeo et al., (2019)	12	10.50 (3.14, 17.86)	13.01	2.80	0.01	25.75
Sarber et al., (2019)	11	7.08 (2.75, 11.42)	7.33	3.20	< 0.01	74.25
Over subgroup	23	7.96 (4.23, 11.70)	9.14	4.18	< 0.01	100
Heterogeneity: Q (1) = 0.62, I^2 = 0%, p = 0.43 Tau^2 = 0						
Scoliosis-absent						
Sarber et al., (2019)	2	3.35	346			

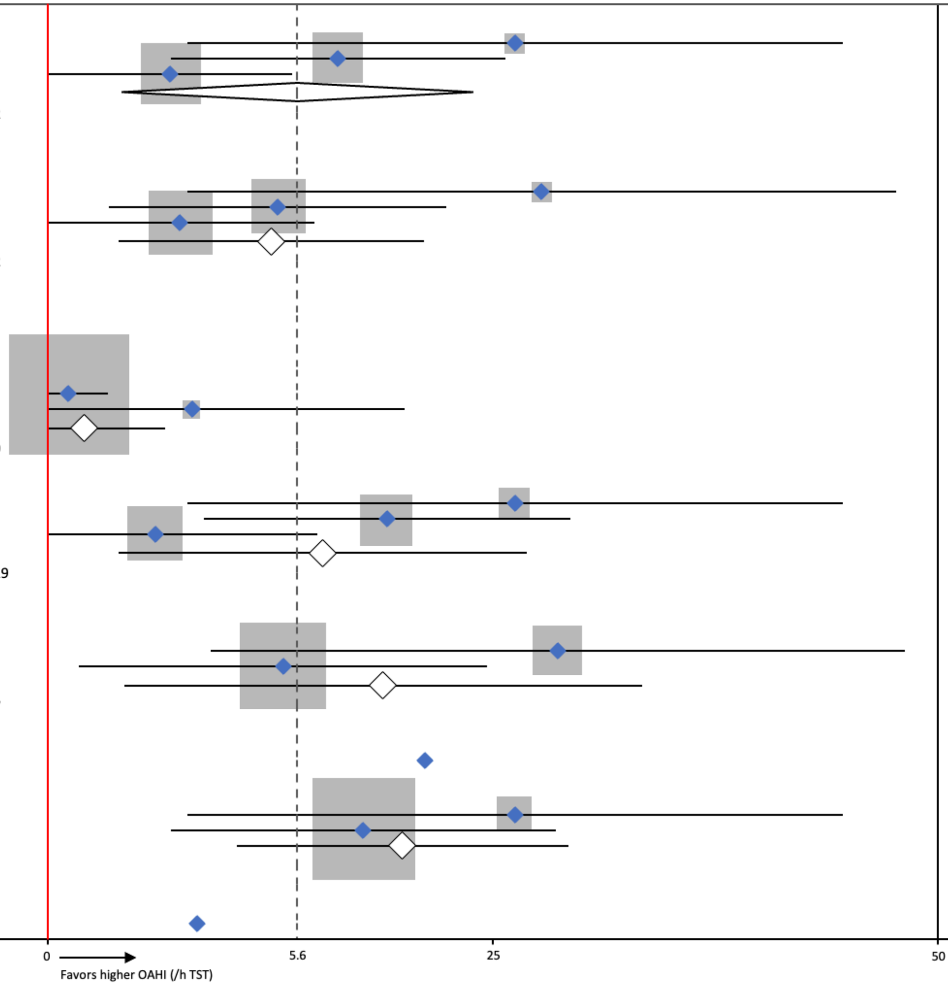

Blue diamond squares indicate mean with confidence interval of 95% (95% CI) in each study. White diamond squares indicate effect size (i.e., ES, pooled mean) after meta combination, which in the total group the width of the diamond and in the subgroups the error bars represent 95% CI. The size of the grey square indicates the relative weight of the study on the combined ES. The arrows intersect with the Y-axis indicate less than the minimum or more than the maximum of diagrammatic range (0 to 50). The grey dotted line indicates ES of RTT total group as a reference value for ES in subgroups. The red dashed line indicates the normative average value and red solid lines indicate the range of normative value, which in overall TD population was value, which in overall TD population was 0 ± 0 /h TST, in <5 years old TD individuals 0 ± 0 /h TST and in >5 years old TD individuals 0 ± 0 /h TST.

Supplementary 2: Figure 11. Forest plots of sleep respiratory in RTT — oxygen desaturation index (ODI) (Part 1)

Study or Subgroup	n	Effect size (95% CI)	SD	Z-Value	p-Value	Weight (random)
**Total group**						
Amaddeo et al., (2019)	12	27.79 (3.56, 52.02)	42.83	2.25	0.02	2.39
Carotenuto et al., (2013)	13	13.09 (10.42, 15.77)	4.92	9.59	< 0.01	73.32
Hagebeuk et al., (2012 a)	10	9.35 (2.53, 16.17)	11.01	2.69	0.01	24.29
**Overall**	**35**	**12.53 (8.75, 16.32)**	**11.42**	**6.49**	**< 0.01**	**100**
Heterogeneity: Q (2) = 2.50, I² = 19.90%, p = 0.29 Tau² = 3.22						
GENE						
MECP2						
Amaddeo et al., (2019)	11	27.41 (0.88, 53.94)	44.90	2.02	0.04	22.46
Hagebeuk et al., (2012 a)	9	9.92 (2.39, 17.45)	11.52	2.58	0.01	77.54
Over subgroup	20	13.85 (0, 28.15)	32.60	1.90	0.06	100
Heterogeneity: Q (1) = 1.54, I² = 35.27%, p = 0.21 Tau² = 53.94						
CDKL5						
No available data						
AGE						
<5 years old						
Hagebeuk et al., (2012 a)	4	6.65	9.77			
>5 years old						
Amaddeo et al., (2019)	12	27.79 (3.56, 52.02)	42.83	2.25	0.02	26.96
Hagebeuk et al., (2012 a)	6	11.15 (1.31, 20.99)	12.30	2.22	0.03	73.04
Over subgroup	18	15.64 (1.16, 30.11)	31.33	2.12	0.03	100
Heterogeneity: Q (1) = 1.56, I² = 35.70%, p = 0.21 Tau² = 49.44						
CLINICAL FEATURES						
Epliespy-present						
Amaddeo et al., (2019)	11	30.14	44.1			
Epliespy-absent						
Amaddeo et al., (2019)	1	2.00	-			
Scoliosis-preset						
Amaddeo et al., (2019)	12	27.79	42.83			
Scoliosis-absent						
No available data						

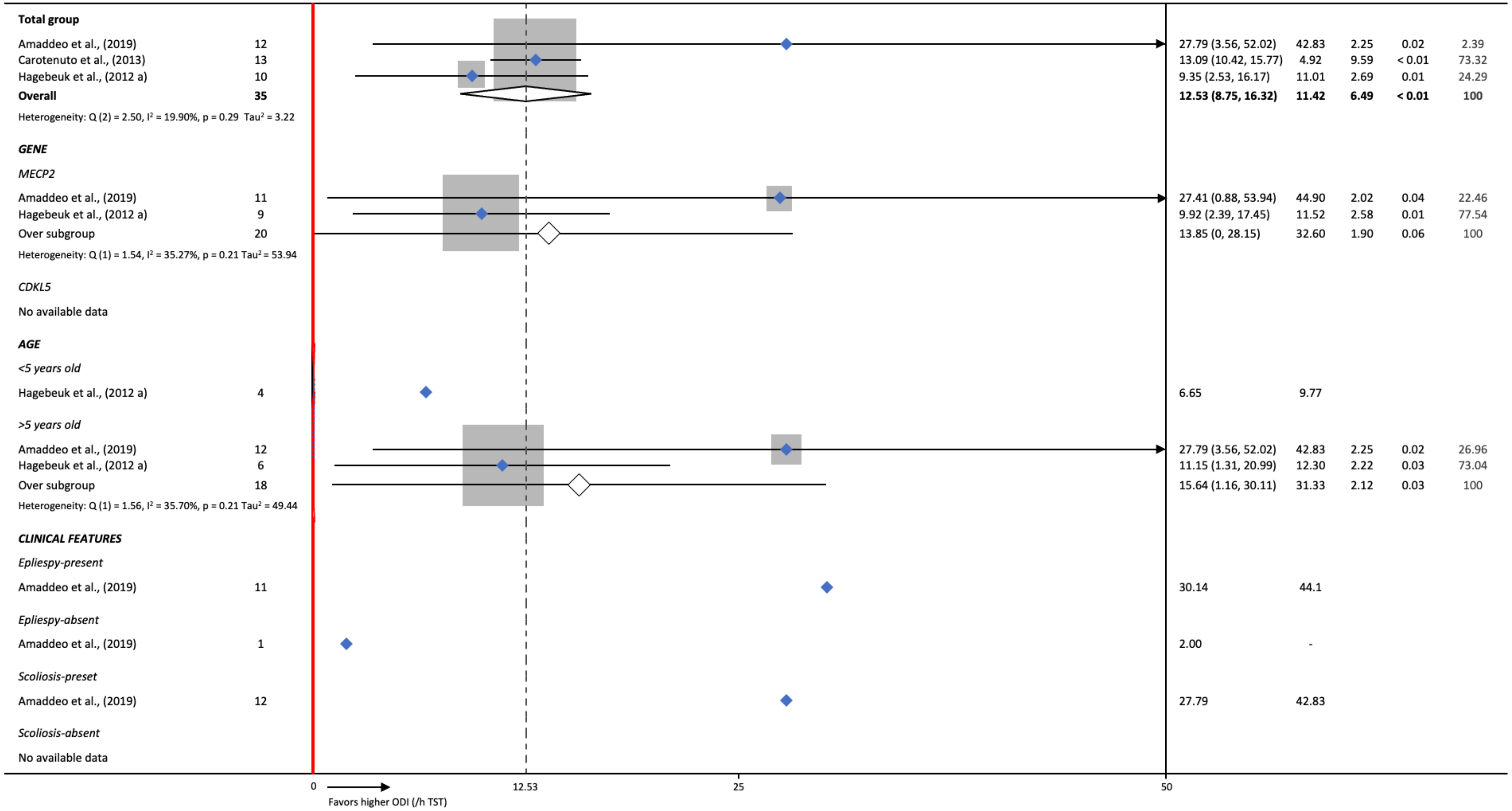

Blue diamond squares indicate mean with confidence interval of 95% (95% CI) in each study. White diamond squares indicate effect size (i.e., ES, pooled mean) after meta combination, which in the total group the width of the diamond and in the subgroups the error bars represent 95% CI. The size of the grey square indicates the relative weight of the study on the combined ES. The arrows intersect with the Y-axis indicate less than the minimum or more than the maximum of diagrammatic range (0 to 50). The grey dotted line indicates ES of RTT total group as a reference value for ES in subgroups. The red dashed line indicates the normative average value and red solid lines indicate the range of normative value, value, which in overall TD population was 0.05 ± 0.05 /h TST, in <5 years old TD individuals 0.1 ± 0 /h TST and in >5 years old TD individuals 0.02 ± 0.05 /h TST.

Supplementary 2: Figure 12. Forest plots of sleep respiratory in RTT — mean oxygen saturation (%) (SpO2% mean) (Part 1)

Study or Subgroup	n	Effect size (95% CI)	SD	Z-Value	p-Value	% weight (random)
**Total group**						
Amaddeo et al., (2019)	12	96.58 (95.37, 97.80)	2.15	155.51	< 0.01	17.55
Carotenuto et al., (2013)	13	93.66 (92.48, 94.84)	2.17	155.81	< 0.01	17.96
Hagebeuk et al., (2012 a)	10	95.41 (93.89, 96.93)	2.45	123.15	< 0.01	14.65
Hagebeuk et al., (2012b)	3	96.30 (95.71, 96.89)	0.52	321.00	< 0.01	24.14
Aldrich et al., (1990)	4	96.50 (96.10, 96.90)	0.41	470.73	< 0.01	25.70
**Overall**	**42**	**95.80 (94.94, 96.65)**	**2.83**	**219.68**	**< 0.01**	**100**
Heterogeneity: Q (4) = 21.50, I^2 = 81.39%, p < 0.01 Tau^2 = 0.70						
GENE						
MECP2						
Amaddeo et al., (2019)	11	96.45 (95.15, 97.76)	2.21	144.92	< 0.01	57.12
Hagebeuk et al., (2012 a)	9	95.17 (93.56, 96.78)	2.47	115.59	< 0.01	42.88
Over subgroup	20	95.90 (94.66, 97.15)	2.84	150.86	< 0.01	100
Heterogeneity: Q (1) = 1.47, I^2 = 32.07%, p = 0.23 Tau^2 = 0.26						
CDKL5						
Hagebeuk et al., (2012b)	4	96.30	0.52			
AGE						
<5 years old						
Hagebeuk et al., (2012 a)	4	95.50 (93.16, 97.82)	2.36	80.86	< 0.01	4.29
Aldrich et al., (1990)	2	96.25 (95.76, 96.74)	0.35	385.00	< 0.01	95.71
Over subgroup	6	96.22 (95.74, 96.70)	2.84	393.40	< 0.01	100
Heterogeneity: Q (1) = 0.39, I^2 = 0%, p = 0.53 Tau^2 = 0						
>5 years old						
Amaddeo et al., (2019)	12	96.58 (95.37, 97.80)	2.15	155.51	< 0.01	13.36
Hagebeuk et al., (2012 a)	6	95.35 (93.17, 97.53)	2.73	85.66	< 0.01	4.16
Aldrich et al., (1990)	2	96.75 (96.26, 97.24)	0.35	387.00	< 0.01	82.48
Over subgroup	20	96.70 (96.22, 97.11)	1.02	425.78	< 0.01	100
Heterogeneity: Q (2) = 1.53, I^2 = 0%, p = 0.47 Tau^2 = 0						
CLINICAL FEATURES						
Epliespy-present						
Amaddeo et al., (2019)	11	96.45 (95.15, 97.76)	2.21	144.92	< 0.01	6.07
Hagebeuk et al., (2012b)	3	96.30 (95.71, 96.89)	0.52	321.00	< 0.01	29.90
Aldrich et al., (1990)	4	96.50 (96.1, 96.90)	0.41	470.73	< 0.01	64.03
Over subgroup	18	96.44 (96.12, 96.76)	0.70	587.90	< 0.01	100
Heterogeneity: Q (2) = 0.30, I^2 = 0%, p = 0.86 Tau^2 = 0						
Epliespy-absent						
Amaddeo et al., (2019)	1	98.00	-			
Scoliosis-present						
Amaddeo et al., (2019)	12	96.58	2.15			
Scoliosis-absent						
No available data						

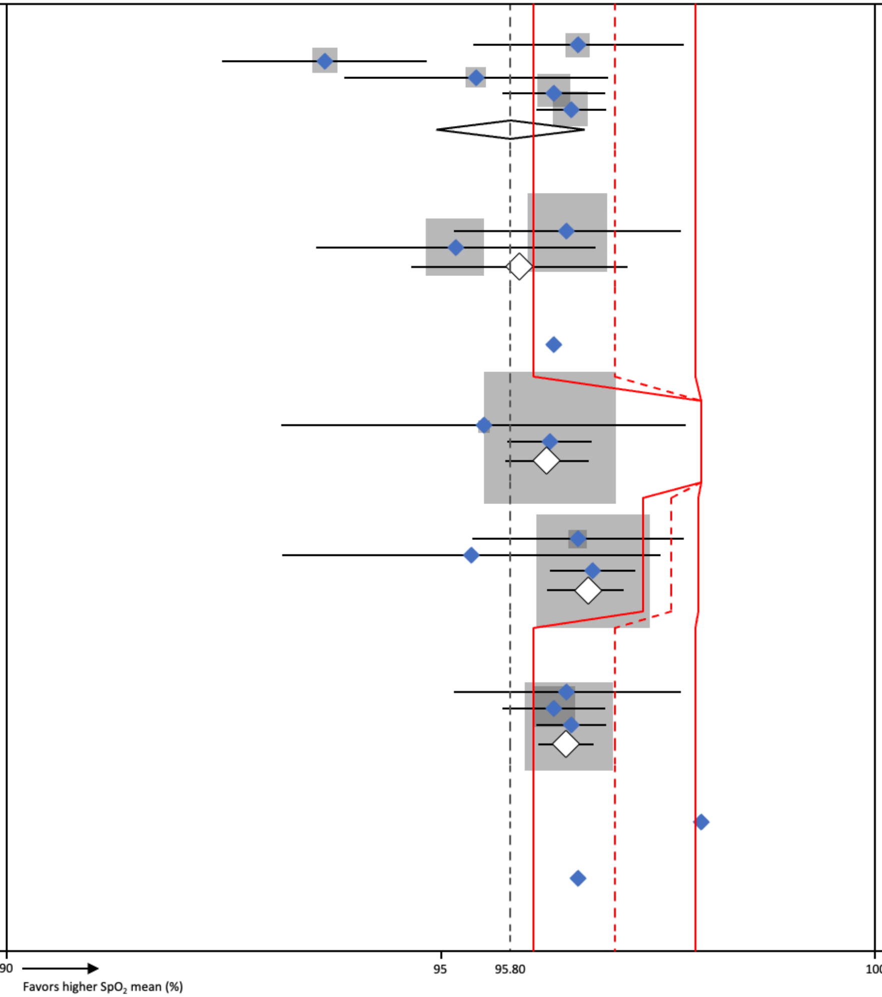

Blue diamond squares indicate mean with confidence interval of 95% (95% CI) in each study. White diamond squares indicate effect size (i.e., ES, pooled mean) after meta combination, which in the total group the width of the diamond and in the subgroups the error bars represent 95% CI. The size of the grey square indicates the relative weight of the study on the combined ES. The arrows intersect with the Y-axis indicate less than the minimum or more than the maximum of diagrammatic range (0 to 100 %). The grey dotted line indicates ES of RTT total group as a reference value for ES in subgroups. The red dashed line indicates the normative average value and red solid lines indicate the range of normative value, which in overall TD population was 97.00 ± 0.94 %, in <5 years old TD individuals 98.00 ± 0.00 % and in >5 years old TD individuals 97.65 ± 0.32 %.

Supplementary 2: Figure 13. Forest plots of sleep respiratory in RTT — minimal oxygen saturation (%) (SpO2% nadir) (Part 1)

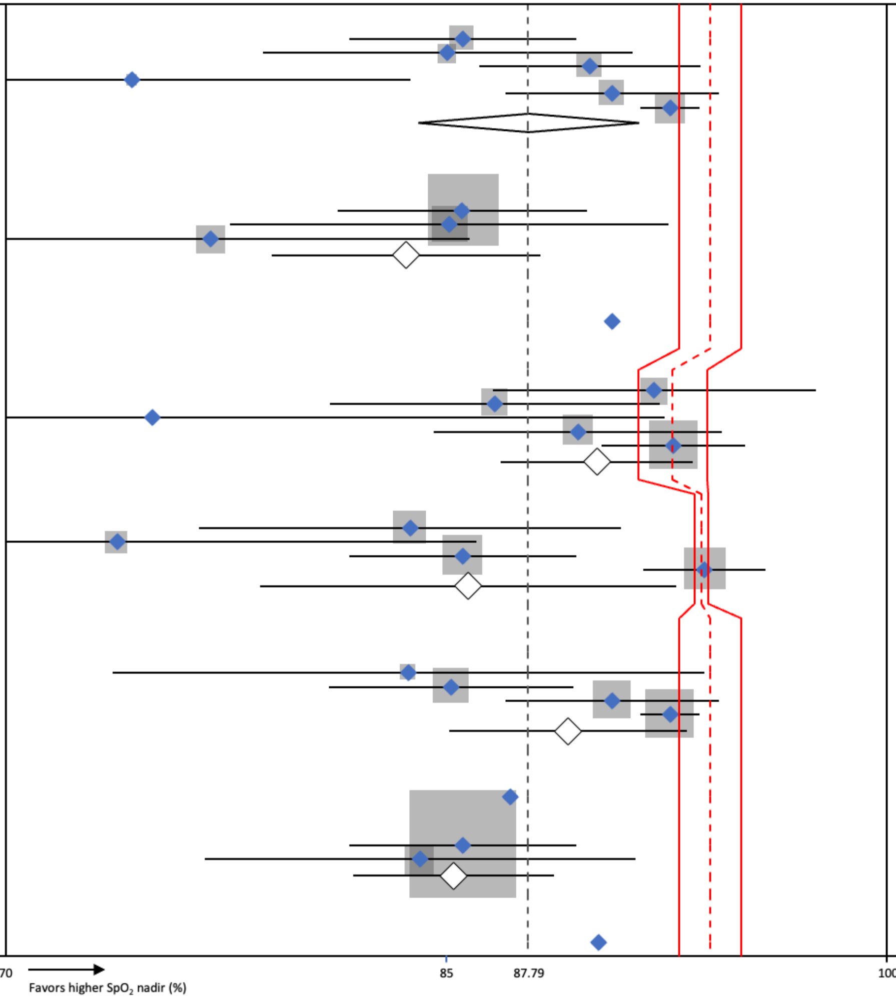

Study or Subgroup	n	Effect size (95% CI)	SD	Z-Value	p-Value	% weight (random)
Total group						
Amaddeo et al., (2019)	12	85.58 (81.71, 89.45)	6.84	43.33	< 0.01	18.13
Sarber et al., (2019)	13	85.06 (78.77, 91.35)	11.58	26.48	< 0.01	13.66
Bassett et al., (2016)	11	89.91 (86.14, 93.68)	6.38	46.74	< 0.01	18.32
Hagebeuk et al., (2012 a)	10	74.30 (64.80, 83.80)	15.33	15.33	< 0.01	9.06
Hagebeuk et al., (2012b)	3	90.67 (87.03, 94.30)	3.21	48.85	< 0.01	18.56
Aldrich et al., (1990)	4	92.63 (91.62, 93.64)	1.03	179.86	< 0.01	22.27
**Overall**	**53**	**87.79 (84.10, 91.49)**	**13.72**	**46.60**	**< 0.01**	**100**
Heterogeneity: Q (5) = 31.17, I^2 = 83.96%, p < 0.01 Tau^2 = 15.68						
GENE						
MECP2						
Amaddeo et al., (2019)	11	85.55 (81.31, 89.79)	7.17	39.55	< 0.01	52.10
Sarber et al., (2019)	11	85.11 (77.65, 92.57)	12.62	22.37	< 0.01	26.93
Hagebeuk et al., (2012 a)	9	77.00 (68.18, 85.82)	13.50	17.11	< 0.01	20.97
Over subgroup	31	83.64 (79.06, 88.22)	13.01	35.78	< 0.01	100
Heterogeneity: Q (2) = 3.00, I^2 = 33.42%, p = 0.22 Tau^2 = 5.80						
CDKL5						
Hagebeuk et al., (2012b)	3	90.67	3.21			
AGE						
<5 years old						
Sarber et al., (2019)	2	92.10 (86.61, 97.59)	3.96	32.89	< 0.01	19.73
Bassett et al., (2016)	6	86.67 (81.06, 92.27)	7.00	30.31	< 0.01	19.27
Hagebeuk et al., (2012 a)	4	75.00 (57.56, 92.44)	17.80	8.43	< 0.01	3.26
Hagebeuk et al., (2012b)	2	89.50 (84.60, 94.40)	3.54	35.80	< 0.01	22.23
Aldrich et al., (1990)	2	92.75 (90.30, 95.20)	1.77	74.20	< 0.01	35.49
Over subgroup	16	90.15 (86.87, 93.42)	6.68	53.97	< 0.01	100
Heterogeneity: Q (4) = 7.93, I^2 = 49.53%, p = 0.09 Tau^2 = 6.30						
>5 years old						
Sarber et al., (2019)	11	83.78 (76.60, 90.96)	12.15	22.88	< 0.01	23.91
Hagebeuk et al., (2012 a)	6	73.83 (61.64, 86.03)	15.24	11.87	< 0.01	16.37
Amaddeo et al., (2019)	12	85.58 (81.71, 89.45)	6.84	43.33	< 0.01	28.92
Bassett et al., (2016)	5	93.80 (91.71, 95.89)	2.38	87.85	< 0.01	30.8
Over subgroup	34	85.76 (78.66, 92.86)	21.12	23.67	< 0.01	100
Heterogeneity: Q (3) = 25.89, I^2 = 88.41%, p < 0.01 Tau^2 = 41.47						
CLINICAL FEATURES						
Epliespy-present						
Sarber et al., (2019)	8	83.73 (73.66, 93.79)	14.52	16.3	< 0.01	11.17
Amaddeo et al., (2019)	11	85.18 (81.03, 89.33)	7.03	40.21	< 0.01	26.03
Hagebeuk et al., (2012b)	3	90.67 (87.03, 94.30)	3.21	48.85	< 0.01	27.79
Aldrich et al., (1990)	4	92.63 (91.62, 93.64)	1.03	179.86	< 0.01	35.02
Over subgroup	26	89.15 (85.10, 93.21)	10.55	43.09	< 0.01	100
Heterogeneity: Q (3) = 14.96, I^2 = 79.95%, p < 0.01 Tau^2 = 11.96						
Epliespy-absent						
Sarber et al., (2019)	5	87.20	4.86			
Scoliosis-present						
Amaddeo et al., (2019)	12	85.58 (81.71, 89.45)	6.84	43.33	< 0.01	78.25
Sarber et al., (2019)	11	84.13 (76.78, 91.47)	12.43	22.45	< 0.01	21.75
Over subgroup	23	85.27 (81.84, 88.69)	8.38	48.80	< 0.01	100
Heterogeneity: Q (1) = 0.12, I^2 = 0%, p = 0.73 Tau^2 = 0						
Scoliosis-absent						
Sarber et al., (2019)	2	90.20	1.27			

Blue diamond squares indicate mean with confidence interval of 95% (95% CI) in each study. White diamond squares indicate effect size (i.e., ES, pooled mean) after meta combination, which in the total group the width of the diamond and in the subgroups the error bars represent 95% CI. The size of the grey square indicates the relative weight of the study on the combined ES. The arrows intersect with the Y-axis indicate less than the minimum or more than the maximum of diagrammatic range (0 to 100 %). The grey dotted line indicates ES of RTT total group as a reference value for ES in subgroups. The red dashed line indicates the normative average value and red solid lines indicate the range of normative value, which in overall TD population was 94.00 ± 1.05 %, in <5 years old TD individuals 92.41 ± 1.17 % and in >5 years old TD individuals 93.71 ± 0.24.
