# Supplementary material for: A meta-review of standard polysomnography parameters in Rett Syndrome
Source: Front Neurol. 2022 Sep 20;13:963626. doi: 10.3389/fneur.2022.963626 (PMC9530595; doi:10.3389/fneur.2022.963626)
Supplement: Supplementary material S3 — Begg's correlation rank test for publication bias. [file Data_Sheet_3.docx]

**Supplement 3: Begg’s correlation rank test for publication bias**

| **Parameters in meta-analysis** | **number of studies** | **Kendall's score** | **z-value** | **p** |
| --- | --- | --- | --- | --- |
| TST | 7 | 7 | 0.9 | 0.38 |
| SOL | 6 | 9 | 1.5 | 0.13 |
| WASO | 6 | 3 | 0.38 | 0.71 |
| SEI | 9 | -16 | -1.77 | 0.12 |
| N1(%) | 8 | 6 | 0.62 | 0.54 |
| N2(%) | 8 | -2 | -0.37 | 0.90 |
| N3(%) | 10 | 3 | 11.18 | 0.86 |
| REM(%) | 9 | 2 | 0.1 | 0.92 |
| AHI | 6 | 5 | 0.75 | 0.45 |
| SpO2mean (%) | 5 | -4 | -1.22 | 0.46 |
| SpO2nadir (%) | 5 | -13 | -2.63 | **0.02** |
